# Low Temperature Promotes Anthocyanin Biosynthesis and Related Gene Expression in the Seedlings of Purple Head Chinese Cabbage (*Brassica rapa* L.)

**DOI:** 10.3390/genes11010081

**Published:** 2020-01-10

**Authors:** Qiong He, Yanjing Ren, Wenbin Zhao, Ru Li, Lugang Zhang

**Affiliations:** 1 State Key Laboratory of Crop Stress Biology for Arid Areas, College of Horticulture, Northwest A&F University, 3 Taicheng Road, Yangling 712100, China; cuidy1988@163.com (Q.H.);; 2 College of Life Sciences, Northwest A&F University, 3 Taicheng Road, Yangling 712100, China; 3 State Key Laboratory of Vegetable Germplasm Innovation, Tianjin 300192, China

**Keywords:** anthocyanin, Chinese cabbage, gene expression, low temperature, seedling

## Abstract

To elucidate the effect of low temperature on anthocyanin biosynthesis in purple head Chinese cabbage, we analyzed anthocyanin accumulation and related gene expression in the seedlings of purple head Chinese cabbage, white head parent Chinese cabbage, and its purple male parent under a normal 25 °C temperature and a low 12 °C temperature. Anthocyanin accumulation in purple lines was strongly induced by low temperature, and the total anthocyanin content of seedlings was significantly enhanced. In addition, nearly all phenylpropanoid metabolic pathway genes (PMPGs) were down-regulated, some early biosynthesis genes (EBGs) were up-regulated, and nearly all late biosynthesis genes (LBGs) directly involved in anthocyanin biosynthesis showed higher expression levels in purple lines after low-temperature induction. Interestingly, a R2R3-MYB transcription factor (TF) gene ‘*BrMYB2*’ and a basic-helix-loop-helix (bHLH) regulatory gene ‘*BrTT8*’ were highly up-regulated in purple lines after low temperature induction, and two negative regulatory genes ‘*BrMYBL2.1*’ and ‘*BrLBD38.2*’ were up-regulated in the white line. *BrMYB2* and *BrTT8* may play important roles in co-activating the anthocyanin structural genes in purple head Chinese cabbage after low-temperature induction, whereas down-regulation of *BrMYB2* and up-regulation of some negative regulators might be responsible for white head phenotype formation. Data presented here provide new understanding into the anthocyanin biosynthesis mechanism during low temperature exposure in *Brassica* crops.

## 1. Introduction

Anthocyanin is a water-soluble pigment of the flavonoid family in plant secondary metabolism, and is responsible for gorgeous coloration and stress resistance in fruits and vegetables [1]. A high concentration of anthocyanin is nontoxic and possesses as a characteristic of reduction the teratogenic and mutagenic incidence; therefore, anthocyanin is widely used in the food industry [2]. Anthocyanin provides beneficial antioxidant functions in human health, leading to a reduction in the incidence of coronary heart disease and anticancer activity [3].

Anthocyanin biosynthesis is one of the most widely studied secondary metabolic pathways in plants, and the genetic and biochemical study of anthocyanin metabolism focused on the understanding of regulation has generated significant scientific breakthroughs [4]. Anthocyanin biosynthesis mainly includes three steps. The primary phenylpropanoid metabolic pathway provides components for subsequent flavonoid and ligin biosynthesis [5] consisting of phenylalanine ammonia lyase (PAL), cinnamate 4-hydroxylase (C4H), and 4-coumarate: CoA ligase (4CL). PAL, which is regarded as the key rate-limiting enzyme in this step, catalyzes the biotransformation of L-phenylalanine into trans-cinnamic acid, and then it is catalyzed by C4H into *p*-coumaric acid [5]. 4CL catalyzes different hydroxy cinnamic acid into the corresponding thioester [6]. The early biosynthesis pathway produces substrates for flavonols and late anthocyanin biosynthesis, which is involved in many fundamental and biological processes in plants [7]. Chalcone synthase (CHS), chalcone isomerase (CHI), flavanone 3-hydroxylase (F3H), flavanone 3’-hydroxylase (F3’H), and flavonol synthase (FLS) are key members in this step, and are mainly activated by independent co-activator and functionally redundant R2R3-MYB transcription factors (TFs) [4]. CHS catalyzes the condensation of three molecules of malonyl Co-A together with *p*-coumarly Co-A into hydroxychalcone, and stereospecific hydroxychalcone is isomerized into naringenin by CHI or only isomerizes spontaneously at a slow rate [8]. F3H catalyzes naringenin into dihydrokaempferol, while F3’H and F3’5’H catalyze it into dihydroquercetin and dihydromyricetin for the following anthocyanin branches, respectively [9]. The late biosynthesis pathway considered to be a key step includes anthocyanin biosynthesis and modification. Usually, activation of the late biosynthesis pathway requires an MBW ternary complex (MYB-bHLH-WD40, formed by a R2R3-MYB factor, a bHLH factor, and a WD40-repeat factor) [10]. Structural enzymes involved in this step are dihydroflavonol 4-reductase (DFR), anthocyanidin synthase (ANS), UDP-glucosyltransferase (UGT), and acyltransferase (AT) [11]. This complex binds to the promoters of anthocyanin biosynthetic genes and activates their transcriptions during development, or responds to a series of environmental signals [3].

Regulators of anthocyanin biosynthesis contain positive regulators and negative regulators. The regulatory network of protein interactions is usually operated through a positive feedback mechanism to control anthocyanin and pro-anthocyanidin synthesis, including R3- and R2R3-MYB regulators, bHLH factor, WD40 repeats, and their interactions [12]. MYB regulator is a large functional family with diverse functions. In *Arabidopsis*, the early biosynthesis pathway is thought to be controlled by R2R3-MYBs such as AtMYB12, AtMYB11, and AtMYB111; these MYBs share significant structural and functional similarity to target early biosynthesis genes (EBGs) such as *AtCHS*, *AtCHI*, *AtF3H*, and *AtFLS1* [13]. R2R3-MYBs AtPAP1, AtPAP2, AtMYB113, and AtMYB114 mainly participate in MBW complex formation, and over-expression of any of them increases anthocyanin production in a TTG1- and bHLH- cooperative manner [4]. Down-regulation of these four *MYBs* reduces expression of late biosynthesis genes (LBGs, including *F3’H*, *DFR*, *ANS*/*LDOX*, *UFGT*) and anthocyanin content [14]. *TT2*, *TT8*, and *TTG1* genes (encoding R2R3-MYB, bHLH and WD40 repeat proteins, respectively) are formed in the MBW complex to activate proanthocyanidin biosynthesis in *Arabidopsis* seeds [15]. However, bHLH genes *TT8*, *GL3*, and *EGL3* partially play redundant roles in the regulation of anthocyanin biosynthesis in seedlings [12]. In addition, negative regulatory factors also play important roles in affecting anthocyanin biosynthesis. Two R3-MYB TFs ‘MYBL2’ and ‘CPC’ (CAPRICE), and three members of lateral organ boundary domain (LBD) factors ‘LBD37’, ‘LBD38’, and ‘LBD39’ are negative regulators of anthocyanin biosynthesis identified in *Arabidopsis* [16,17,18,19]. AtMYBL2 and AtCPC inhibit anthocyanin accumulation either by repressing the biosynthesis genes or by directly inhibiting the activity of the MBW complexes [17,18]. AtMYBL2 represses expression level of MYB and bHLH genes such as *AtTT8*, *AtPAP1*, and *AtPAP2*, and it occurs predominantly but not exclusively in seeds and vegetative tissues of adult plants, with anthocyanins not accumulated or *TT8* not expressed [16,17]. Interestingly, *AtTT8* positively regulates the expression of *AtMYBL2* [12]. LBD family members ‘AtLBD37’, ‘AtLBD38’, and ‘AtLBD39’, act as negative regulators through suppressing the key regulator genes *AtPAP1* and *AtPAP2* of anthocyanin biosynthesis in *Arabidopsis* [19]. These findings suggest that anthocyanin biosynthesis pathway might be mediated by both activators and repressors.

Furthermore, anthocyanins were largely induced under abnormal stress environments, such as high light, nutrient depletion, or excess and low temperature [20,21,22]. Cold is considered to be an important factor in inducing anthocyanin accumulation, which has been confirmed in strawberry [23], grape [24,25], crabapple [26], peach [27], red orange [28], apple [29,30], and purple kale (*Brassica oleracea* var. *acephala* f. *tricolor*) [31]. Advances in comprehension of anthocyanin metabolism in response to stress and diverse environment have been produced in recent years [32]. One important pathway to understand the role of anthocyanin in stress is to determine how expression of the biosynthetic pathway is operated and which members have participated in this important pathway. Research in model plants indicated that low temperature treatment of *Arabidopsis* seedlings induced a large accumulation of anthocyanins, accompanied by the up-regulation of *AtC4H*, *AtCHS*, *AtCHI*, *AtF3H*, *AtDFR*, *AtANS*, and *AtUFGT* [33]. Similarly, Sun et al. found that low temperature induced promoter demethylation of R2R3-MYB regulator gene *CsAN1* and resulted in up-regulation of LBGs and anthocyanin accumulation in tea [34]. In grape cultivation, low temperature generally promotes anthocyanin accumulation and up-regulation of anthocyanin biosynthesis genes *VvF3H*, *VvPAL*, *VvCHS3*, *VvCHS2*, and *VvLDOX* [24].

Heading Chinese cabbage (*Brassica rapa* L. ssp. *Pekinensis*) is widely cultivated as an economic plant in Asian countries, and heading leaves are usually curly with brilliant white, yellow, or orange color. However, head of the Chinese cabbage phenotype with an anthocyanin-rich character is of interest due to a lack of the novel natural mutant. Purple head in Chinese cabbage leads to a large accumulation of anthocyanins in the heading leaves in winter, and the regulatory mechanism of anthocyanin biosynthesis under low temperature conditions in *Brassica* vegetable crops is to date poorly studied. Hence, the new purple head Chinese cabbage provides an excellent opportunity to further investigate the mechanism of anthocyanin biosynthesis under low temperature conditions. In addition, genes usually have one copy in *Arabidopsis* genome, whereas multiple copies of anthocyanin biosynthesis gene of *Brassica* plants were mainly produced during whole genome duplication and retained synteny with their orthologs from *Arabidopsis* [35]. Some LBGs and positive regulatory genes seem to have fewer than three copies as a result of gene loss during subsequent whole genome duplication, whereas more PMGs, EBGs, and negative regulatory genes are retained [35]. Ahmed et al. only characterized four *ANSs* and 12 *DFRs* of *B. rapa* and investigated their association with pigment formation in cold and freezing tolerance [36,37]; *BrANS1*, *BrANS3*, *BrDFR2*, *BrDFR4*, *BrDFR8*, and *BrDFR9* only showed very high responses to cold stress in pigmented *B. rapa* samples, while *BrANS2*, *BrDFR1*, *BrDFR3*, *BrDFR5*, *BrDFR6*, and *BrDFR10* responded to cold and freezing stress treatments [36,37]. This information implied that perhaps there are a great number of PMGs, EBGs, LBGs and regulatory genes in purple head Chinese cabbage genome that may affect anthocyanin accumulation, and there are doubts over whether all these genes are involved in the purple trait formation and how they are expressed in purple head Chinese cabbage. Hence, to mine meaningful information of anthocyanin biosynthesis under low temperature conditions, we compared the anthocyanin accumulation and expression characters of related biosynthesis genes in seedlings of three genotypes of Chinese cabbage. A total of 86 anthocyanin biosynthetic genes identified in *B. rapa* as 51 orthologs of anthocyanin biosynthetic genes in *Arabidopsis* were detected to supply evidence about the effect of low temperature on anthocyanin biosynthesis in purple head Chinese cabbage.

## 2. Materials and Methods

### 2.1. Plant Materials and Growth Conditions

**Figure 1:**
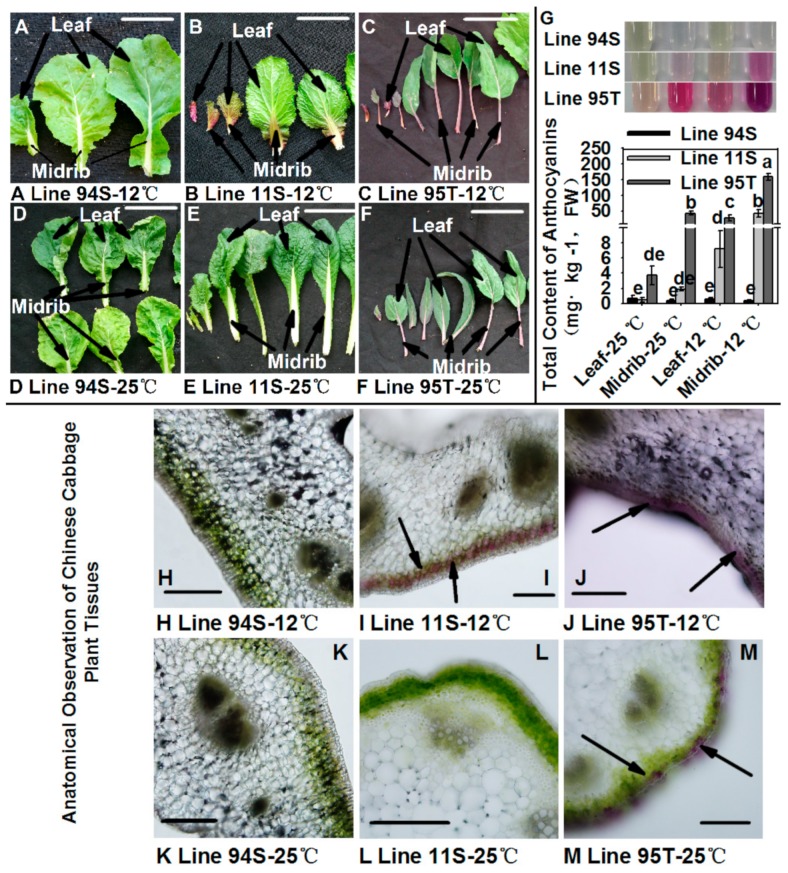
Seedlings of Chinese cabbages under 25 °C and 12 °C conditions. Plants of three lines were grown under a 12-h light photoperiod at 25 °C, 125 mmol m^−2^s^−1^; 28-day seedlings were put at 12 °C for 15 days, and seedlings that stayed at 25 °C with same light condition were treated as the control. (**A**–**F**) appearance of individual seedling leaves picked from seedlings of three lines; (**G**) the extract solution of samples (the solution was 1% hydrochloric acid water solution) and the total anthocyanin content of samples; (**H**–**M**) anatomical observation of leaf midrib tissues. The scale bar is 5 cm and 200 μm in (**A**–**F**) and (**H**–**M**), respectively. Arrowheads indicate anthocyanin accumulation.

A pure heading Chinese cabbage Line11S91 (11S) with a stable inheritance of purple head, an inbred Line94S19 (94S, a female parent of 11S) with a white head, and an inbred line of flowering Chinese cabbage Line95T2 (95T, the male parent of 11S) with deep purple leaves and stems were used in this study. Plants of three lines were grown in black plastic bowls (10 × 10 cm) containing peat, vermiculite, and perlite at a volume ratio of 3:1:1 under a 12 h light/12 h dark photoperiod at 25 °C, 125 mmol m^−2^s^−1^. To study the effect of low temperature on anthocyanin accumulation in Chinese cabbages, 28-day seedlings were put at 12 °C for 15 days on a 12 h light/12 h dark photoperiod 125 mmol m^−2^s^−1^; seedlings which stayed at 25 °C with same light condition were treated as the control. Seedling leaves of every line were divided into two parts: the midribs and the leaves (Figure 1A–F). Samples were collected, frozen immediately in liquid nitrogen, and stored at −80 °C in a refrigerator (Sanyo, Osaka, Japan) until RNA isolation and anthocyanin extraction. Each sample was analyzed in triplicate, and three biological replicates were conducted.

### 2.2. Histological Observation of Seedling Leaves

Samples of leaf midrib of three Chinese cabbages treated at room and low temperature were prepared for histological observation by free-hand sectioning as previously described [38]. Samples were investigated with a fluorescent microscope (Olympus, Tokyo, Japan) at a befitting magnification under a bright field.

### 2.3. Total Anthocyanins Analysis

Total anthocyanin extraction and determination were measured using an UV-visible spectroscopy method as previously with a few modifications [39]. Crushed samples (1.0 g) were extracted using the 1% hydrochloric acid water solution (10 mL), and measured at λ_max_ 530 nm and 700 nm using a UV–visible spectrophotometer (Thermo Fisher Scientific, Wilmington, USA) in a 0.2-M potassium chloride buffer (pH 1.0) and a 0.45-M sodium acetate buffer (pH 4.5), respectively. These dilutions were equilibrated for 3 h at 4 °C in the dark. Total anthocyanins were calculated using previous equation [40]. Results are expressed as average of three biological replicates.

### 2.4. Identification of Anthocyanin Biosyntheis Genes and Quantitative Real-Time PCR (qRT-PCR) Analysis

Total RNA extraction, cDNA synthesis, and the qRT-PCR reactions in IQ5 optical system (Bio-Rad, USA) were performed as described previously [39]. The synthetic cDNA was diluted to 50 ng μL^−1^ with double-distilled water using a microspectrophotometer (Thermo Fisher Scientific, Wilmington, DE, USA). The qRT-PCR was conducted with a three-step cycling procedure by following the instruction of SYBR Premier Ex Taq II mix (Takara, Kusatsu, Japan). To identify anthocyanin biosynthesis genes in Chinese cabbage, the reported anthocyanin biosynthesis gene sequences in *Arabidopsis thaliana* were downloaded from the TAIR database (https://www.arabidopsis.org/) and used as a query to perform BLASTP searching in the whole genome of Chinese cabbage in BRAD database (http://brassicadb.org/brad/). The primers (listed in Tables S2 and S3) for the qRT-PCR were designed by Primer Premier 5.0 (Premier, Vancouver, BC, Canada), and their specificity was checked by NCBI Primer BLAST (http://www.ncbi.nlm.nih.gov/tools/primer-blast/index.cgi?LINK_LOC=BlastHome) and the *Brassica* database (BRAD, http://brassicadb.org/brad/blastPage.php). All qRT-PCR data were normalized using the cycle threshold (CT) value corresponding to *BrEF-1-α*. Melting curve analysis of samples revealed that only one product was produced from each primer pair, which confirmed specific amplification. All reactions were manipulated in three replicates. The relative expression levels of these target genes were handled by the 2^−∆∆CT^ method [41] in IQ5 software.

### 2.5. Clustering Analysis and Interaction Network

Gene expressions that indicated statistically significant changes about anthocyanin accumulation in three Chinese cabbages were grouped using a two-way hierarchical clustering methodology by the PermutMatrix software [42]. The Euclidean distance and Ward’s method were used for aggregation. The interaction network of anthocyanin biosynthesis genes in *Arabidopsis* was produced by the STRING software [43], and the putative interaction networks in Chinese cabbage were created according to the corresponding homologs of *Arabidopsis*.

### 2.6. Statistical Analysis

Data were subjected to a one-way analysis of variance (ANOVA) using SPSS version 13.0 (SPSS, Chicago, IL, USA), and a Duncan’s multiple-range test was determined for the data at the 0.05 confidence level. A bivariate analysis of the Pearson correlation coefficient was calculated in a two-tailed test.

## 3. Results

### 3.1. Low Temperature Induces Anthocyanin Accumulation

Compared to Line94S, both leaves and midribs of Line11S and Line95T exhibited a purple color after 12 °C treatment, along with red color in the hydrochloric acid aqueous extracts (Figure 1A–C,G). Further microscopic observation of midrib section from seedling leaves of Line11S and Line95T illustrated that purple pigments massively accumulated in two cell layers under the midrib epidermis cell after low temperature treatment (Figure 1I,J), which was in accordance with the red color appearance in hydrochloride aqueous extracts (Figure 1G). In contrast, green chlorophyll layers were observed in Line94S at the same location, and extracts showed no color change (Figure 1H,K,G). Total anthocyanin contents of seedling midrib in Line11S and Line95T were significantly enhanced and reached up to 42.393 and 158.305 mg kg^−1^, respectively (Figure 1G). Comparatively, total anthocyanin contents of them in the leaf part were lower than in midrib (Figure 1G). Anthocyanins in purple lines are strongly induced to accumulate after low temperature treatment, especially in the midribs.

### 3.2. Low Temperature Significantly Down-Regulated Most Phenylpropanoid Metabolic Pathway Genes (PMPGs)

**Figure 2:**
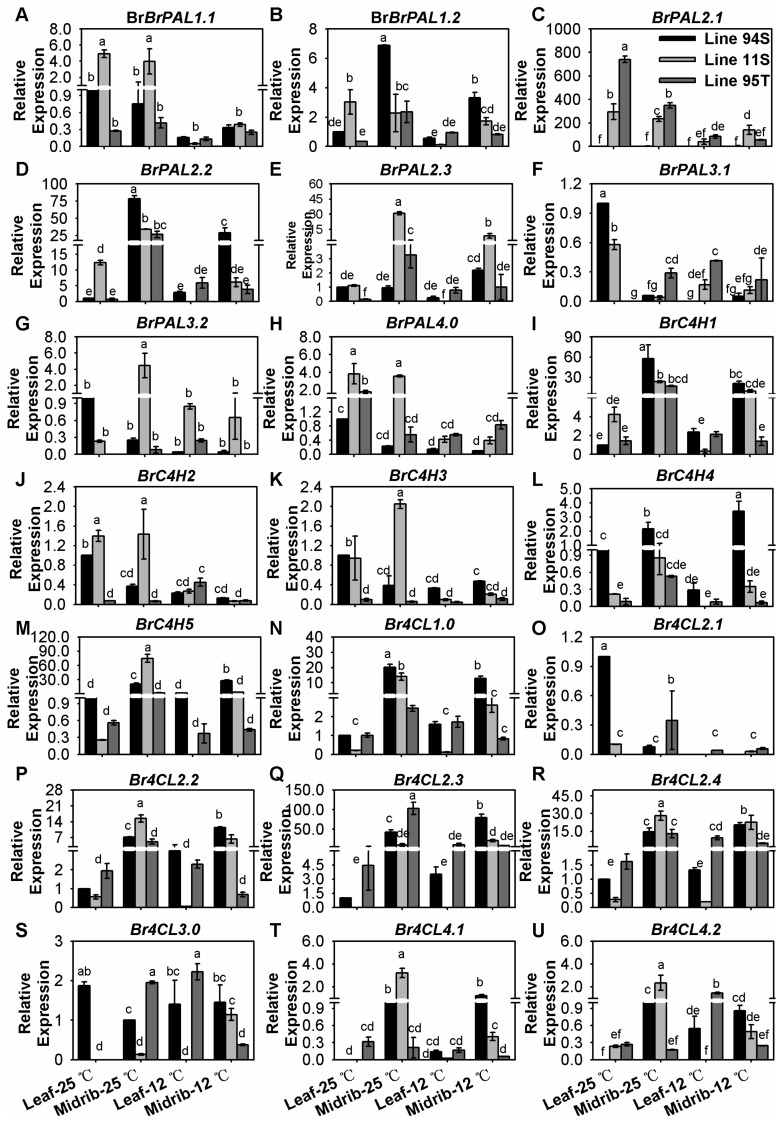
Expression of phenylpropanoid metabolic pathway genes (PMPGs) in the seedlings of three Chinese cabbages under low temperature treatment. (**A**–**H**) relative expression of *BrPALs*; (**I**–**M**) relative expression of *BrC4Hs*; (**N**–**U**) relative expression of *Br4CLs*. Plants of three lines were grown under a 12-h light photoperiod at 25 °C, 125 mmol m^−2^s^−1^; 28-day seedlings were treated at 12 °C for 15 days, and seedlings that stayed at 25 °C with same light condition were treated as the control. Seedling leaves were divided into the midribs and the leaves, and leaves of Line94S at 25 °C served as the control. Values are presented as means ± SD (*n* = 3). The different letters above each column are significantly different at *p* < 0.05 by Duncan’s test.

A total of 21 PMPGs were investigated, including eight *BrPALs*, five *BrC4Hs*, and eight *Br4CLs*. These genes showed different expression levels in leaves and midribs of the seedlings in three genotypes of Chinese cabbage (Figure 2 and Table S1). All *BrPALs* were down-regulated in both leaves and midribs of three lines under 12 °C treatment, with the high expression decreasing to about one-fold in the midrib and leaves (Figure 2A–H and Table S1). These *BrPALs* also displayed a tissue-specific character. For example, *BrPAL1.2*, *BrPAL2.2*, and *BrPAL2.3* showed higher expression in midribs than in leaves at both two conditions, whereas *BrPAL2.1* and *BrPAL3.1* showed the opposite expression character; *BrPAL1.1*, *BrPAL3.2*, and *BrPAL4.0* showed similar expression levels between leaves and midribs, respectively (Figure 2A–H and Table S1). Notably, only *BrPAL2.1*, *BrPAL2.2*, and *BrPAL2.3* were highly expressed in three lines, and ‘*BrPAL2.1*’ showed novel high expression level in two tissues in two purple Chinese cabbages (Figure 2C–E and Table S1). The expression character of *BrPAL2.1* was accordance with the total anthocyanin content, with highest level in Line95T, followed by Line11S and Line94S in both leaves and midribs under two conditions, respectively (Figure 2C and Table S1). *BrPAL1.1*, *BrPAL2.3*, and *BrPAL3.2* expressed with higher levels in Line11S than in Line94S and Line95T; *BrPAL1.2* and *BrPAL2.2* were expressed with higher levels in Line94S than in Line11S and Line95T (Figure 2A–H and Table S1). Generally, low temperature treatment reduced expression of most *BrC4Hs* to varying degrees. Only *BrC4H1.0* and *BrC4H5.0* were highly expressed in three lines, and low temperature treatment produced a minor increase of expression in them (Figure 2I–M and Table S1). In addition, *BrC4H4* is moderately up-regulated in the leaves in Line94S under low temperature (Figure 2L). Although cold temperatures moderately down-regulated *Br4CLs*, they are less sensitive to low temperature than *BrPALs* and *BrC4Hs* (Figure 2 and Table S1). Notably, *Br4CLs* expressed higher expressions in midribs than in leaves except for *Br4CL2.1* and *Br4CL3.0*. *Br4CL1.0*, *Br4CL2.2*, *Br4CL2.3* and *Br4CL2.4* were highly expressed in midribs of three lines (Figure 2N–U and Table S1). In *Arabidopsis*, *At4CL1*, *At4CL2*, and *At4CL4* are more closely to each other than *At4CL3*, and these four genes mainly participated in lignin biosynthesis [6]. *At4CL3* plays a distinct role in flavonoid metabolism, and *At4CL1* also showed similar function in this pathway [6]. Apart from flavonoid metabolism, these *Br4CLs* with high expressions might play redundant roles of lignin biosynthesis in the midrib. Only *BrPAL2.1* was especially expressed in purple head Chinese cabbage and its purple donation, and all these investigated PMPGs were expressed more or less in white Line94S (Figure 2 and Table S1). This implied that the initial phenylpropanoid metabolic pathway was essential biological process in all three Chinese cabbages, but they might not be the key step affecting the final anthocyanin accumulation in purple Chinese cabbage.

### 3.3. Low Temperature Moderately Up-Regulated the EBGs for Subsequent Anthocyanin Biosynthesis

**Figure 3:**
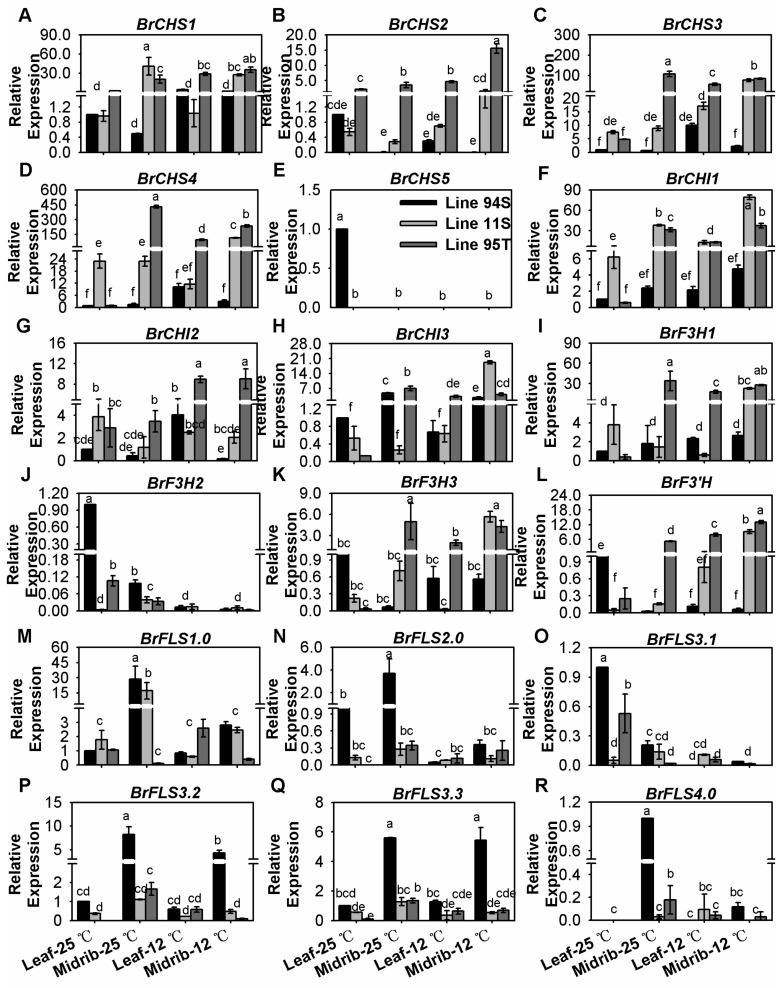
Expression of early biosynthesis genes (EBGs) in the seedlings of three Chinese cabbages under low temperature treatment. (**A**–**E**) relative expression of *BrCHSs*; (**F**–**H**) relative expression of *BrCHIs*; (**I**–**K**) relative expression of *BrF3Hs*; (**L**) relative expression of *BrF3’H*; (**M**–**R**) relative expression of *BrFLSs*. Plants of three lines were grown under a 12-h light photoperiod at 25 °C, 125 mmol m^−2^s^−1^; 28-days seedling were treated at 12 °C for 15 days, and the seedlings that stayed at 25 °C with same light condition were treated as the control. Seedling leaves were divided into the midribs and the leaves, and leaves of Line94S at 25 °C served as the control. Values are presented as means ± SD (*n* = 3). The different letters above each column are significantly different at *p* < 0.05 by Duncan’s test.

In general, low temperature moderately induced the second early biosynthesis pathway for following anthocyanin biosynthesis, whereas members such as *BrFLSs* for flavonol biosynthesis were down-regulated (Figure 3 and Table S1). Except for *BrCHS5* was scarcely expressed, expression degrees of all *BrCHSs* were appropriately up-regulated by a small extent after low temperature induction (Figure 3A–E and Table S1). *BrCHS4* displayed the highest expression level, followed by *BrCHS3*, *BrCHS1*, and *BrCHS2*, and *BrCHS4*, *BrCHS3* and *BrCHS2* showed similar transcriptional characters to total anthocyanin content (Figure 1G and Figure 3A and Table S1). In detail, *BrCHS1.0* only showed a small increase in both treated leaves and midribs in three lines (Figure 3A and Table S1); *BrCHS2.0* was relatively more sensitive to low temperature and increased in leaves and midribs in two purple lines (Figure 3B and Table S1). However, increased expression of *BrCHS3.0* and *BrCHS4.0* was mainly concentrated on the leaf proportion in two purple lines (Figure 3C,D and Table S1). Comparatively, *BrCHIs* displayed higher dose of increase after low temperate treatment, and *BrCHI1.0* and *BrCHI3.0* would be more active to response in cold induction (Figure 3F–H and Table S1). *BrCHI1* displayed the highest expression level, followed by *BrCHI3* (Figure 3F–H and Table S1). Expression characters of *BrF3H1.0*, *BrF3H3.0*, and *BrF3’H* were similar to them, whereas *BrF3H2.0* was scarcely detected in all lines (Figure 3I–L and Table S1). Obviously, *BrF3H1.0* showed much higher expression than *BrF3H3.0*, which indicated it might be more active in the early biosynthesis process. Interestingly and similarly to PMPGs, all *BrFLSs* were down-regulated in three Chinese cabbages; *BrFLSs* had higher expression in white head Chinese cabbage than in purple lines, and *BrFLS1.0* displayed the highest expression (Figure 3M–R and Table S1). FLSs transform substrates into colorless flavonols, which are competitive to DFR in the following anthocyanin production branch [44]. It implied that BrFLSs compete substrates with DFR in head Chinese cabbage, and that which is superior might decide the next biosynthesis step.

### 3.4. Key LBGs and Transport Genes Were Highly Induced under Low-Temperature Condition

**Figure 4:**
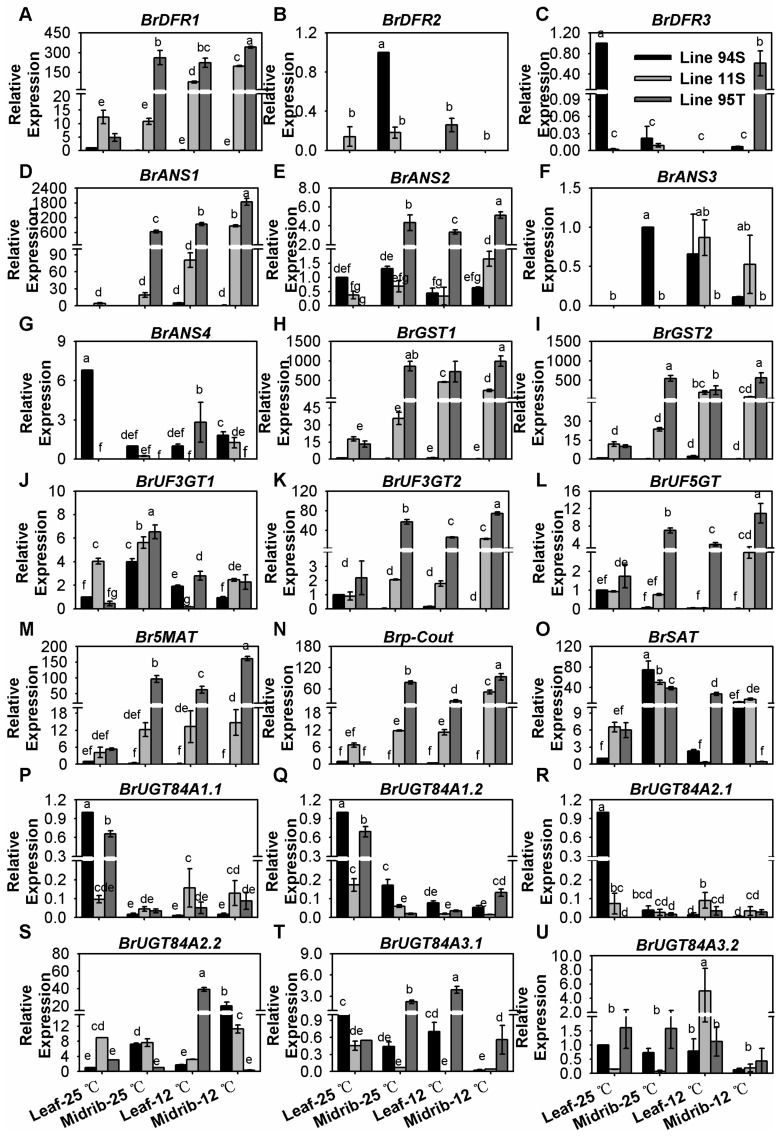
Expression of late biosynthesis genes (LBGs) and transport *BrGSTs* in the seedlings of three Chinese cabbages under low temperature treatment. (**A**–**C**) relative expression of *BrDFRs*; (**D**–**G**) relative expression of *BrANSs*; (**H**,**I**) relative expression of *BrGSTs*; (**J**–**L**) relative expression of *BrUGTs*; (**M**–**O**) relative expression of *BrATs*; (**P**–**U**) relative expression of *BrUGT84As*. Plants of three lines were grown under a 12-h light photoperiod at 25 °C, 125 mmol m^−2^s^−1^; 28-day seedlings were treated at 12 °C for 15 days, and the seedlings that stayed at 25 °C with same light condition were treated as the control. Seedling leaves were divided into the midribs and the leaves, and leaves of Line94S at 25 °C served as the control. Values are presented as means ± SD (*n* = 3). The different letters above each column are significantly different at *p* < 0.05 by Duncan’s test.

It is noteworthy that *BrDFR1*, *BrANS1*, *BrUF3GT2*, *BrUF5GT*, *BrUF5MAT*, *Brp-Cout*, *BrGST1*, and *BrGST2* in leaves and midribs were scarcely detected in white Line94S, but their expressions were significantly up-regulated after cold induction in two purple lines, especially in the midribs (Figure 4 and Table S1). Though the expression levels of them were similar in 12 °C-treated leaves and midribs in a purple line, they were highly expressed in midribs at room temperature (Figure 4 and Table S1). *BrANS2*, another homolog of *BrANS1*, was tenderly increased about three times in Line95T after cold induction (Figure 4E and Table S1). In contrast, *BrDFR2*, *BrDFR3*, *BrANS3*, and *BrANS4* were scarcely expressed in both treated and untreated samples of three Chinese cabbages (Figure 4B,C,F,G). These results indicated that *BrDFR1*, *BrANS1*, *BrUF3GT2, BrUF5GT, BrUF5MAT, Brp-Cout, BrGST1*, and *BrGST2* were extremely sensitive (especially in leaves) to induction by low temperature to participate in the late anthocyanin biosynthesis and transportation pathway. Comparatively, the low temperature supplied a degree of inhibition of *BrUF3GT1* (Figure 4J). *AtSCPL* (*At2g23000*), which encodes sinapoyl-Glc:anthocyanin acyltransferase, is required for the synthesis of sinapoylated anthocyanins in *Arabidopsis* [45]. *UGT84As*, the sinapic acid:UDP-glucose glucosyltransferase genes, compose a big family and some of them play major roles in sinapoylation of anthocyanin [46]. *AtUGT84A2* has the ability to gather significant source of sinapoyl moieties for anthocyanin modification, and a limited supply of 1-O-sinapoylglucose (produced by *AtUGT84A2*) reduces the content of sinapoylated anthocyanin [46]. In *Arabidopsis*, *AtUGT84A3* tentatively suggests that it has an impact on cell wall-associated 4-coumarate, whereas *AtUGT84A4* would be enhanced under UV-B radiation and is accompanied by a transient increase of sinapoylglucoses and sinapoylmalates [47]. In our reports, *BrSAT* (a homology to *AtSCPL* with similar expression pattern to PMPGs) was highly expressed in both treated and untreated samples in three lines, and it was slightly decreased under the low temperature condition (Figure 4O and Table S1). Only *BrUGT84A2.2* showed sensitive character under low temperature in three Chinese cabbage seedlings; other copies of *BrUGT84A* were with low expression level in both two tissues (Figure 4P–U and Table S1). These results implied that *BrSAT* and *BrUGT84A2.2* might participate in the production of sinapoylated anthocyanins in purple Chinese cabbages.

### 3.5. Effect of Low Temperature on the Expression of Positive Regulatory Genes

**Figure 5:**
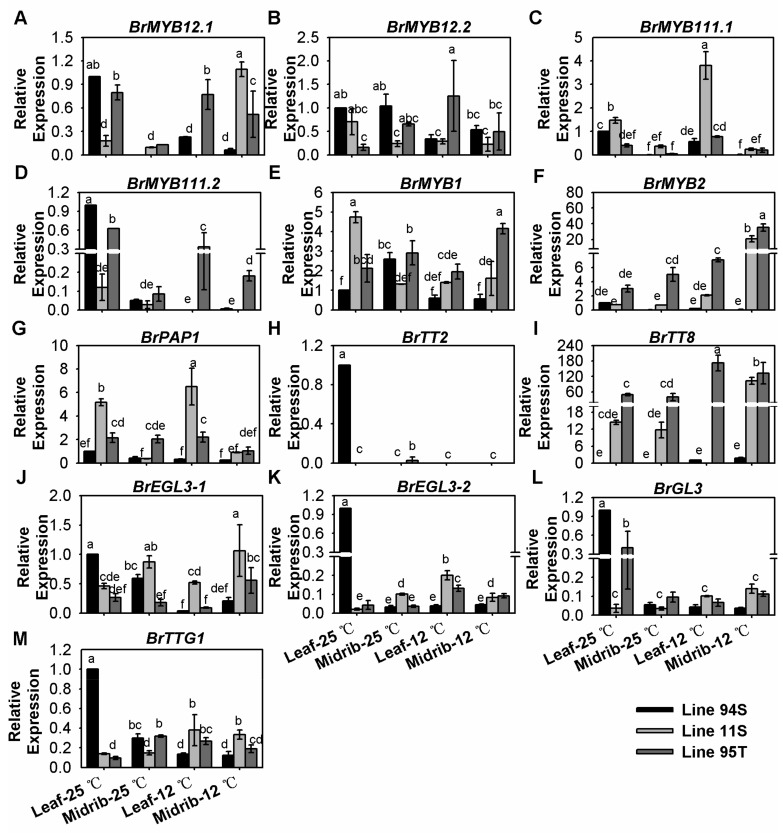
Expression of positive regulatory genes in the seedlings of three Chinese cabbages under low temperature treatment. (**A**–**H**) relative expression of R2R3-MYB regulatory genes; (**I**–**L**) relative expression of bHLH (basic-helix-loop-helix) genes; (**M**) relative expression of *BrTTG1*. Plants of three lines were grown under a 12-h light photoperiod at 25 °C, 125 mmol m^−2^s^−1^; 28-day seedlings were treated at 12 °C for 15 days, and the seedlings that stayed at 25 °C with same light condition were treated as the control. Seedling leaves were divided into the midribs and the leaves, and leaves of Line94S at 25 °C served as the control. Values are presented as means ± SD (*n* = 3). The different letters above each column are significantly different at *p* < 0.05 by Duncan’s test.

*BrMYB12.1* and *BrMYB12.2* are homologies to *AtMYB12*, whereas *BrMYB111.1* and *BrMYB111.2* are homologies to *AtMYB111*. These MYBs showed extremely low expressions in three lines, and the differences were not significant among three lines (Figure 5A–D and Table S1). *BrMYB12.1* showed a slight rise after 12 °C -treatment in midribs in Line11S and Line 95T; *BrMYB111.1* slightly increased about three times in leaves in treated Line11S (Figure 5A,C and Table S1), whereas *BrMYB111.2* showed a tiny increase in the midribs in Line95T (Figure 5C,D and Table S1). Except for *BrMYB2* and *BrTT8*, all positive regulators activated LBGs were lowly expressed in three lines (Figure 5E–M and Table S1). After cold induction, *BrMYB2* was highly expressed in Line95T, and its expression level was higher than in Line11S; however, there was no change of *BrMYB2* expression in Line94S (Figure 5F and Table S1). *BrMYB2* was up-regulated in both leaves and midribs in Lines 11S and 95T after 12 °C-treatment, with the expression increasing from 0.7-fold to 2.1-fold in the leaves and 0.6-fold to 15-fold in the midribs of Line11S, respectively; it increased from 3-fold to 7-fold in the leaves and 4-fold to 31-fold in the midribs in Line95T, respectively (Figure 5F and Table S1). *BrTT8* was induced in two parts after 12 °C -treatment in Line95T, but it was only induced in midrib in Line11S (Figure 5I and Table S1). These results indicated that the R2R3-MYB regulator gene *BrMYB2* and *BrTT8* might be active and induced by low temperature during anthocyanin biosynthesis in purple head Chinese cabbage and its purple trait donor, but *BrMYB2* played a key role in the anthocyanin accumulation. 

### 3.6. Effect of Low Temperature on the Expression of Negative Regulatory Genes

**Figure 6:**
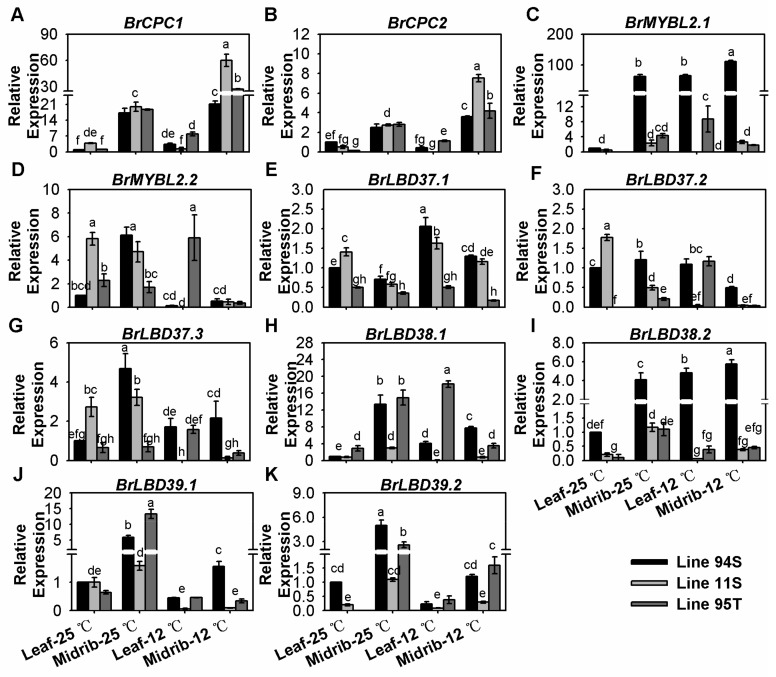
Expression of negative regulatory gene**s** in the seedlings of three Chinese cabbages under low -temperature treatment. (**A**–**D**) relative expression of R3-MYB regulatory genes; (**E**–**K**) relative expression of LBD (LATERAL ORGAN BOUNDARY DOMAIN) genes. Plants of three lines were grown under a 12-h light photoperiod at 25 °C, 125 mmol m^−2^s^−1^; 28-day seedlings were treated at 12 °C for 15 days and the seedlings that stayed at 25 °C with same light condition were treated as the control. Seedling leaves were divided into the midribs and the leaves, and leaves of Line94S at 25 °C served as the control. Values are presented as means ± SD (*n* = 3). The different letters above each column are significantly different at *p* < 0.05 by Duncan’s test.

Two *BrCPCs* and *BrLBD37*.1 were induced by low temperature in all lines (Figure 6A,B,E and Table S1). Notably, *BrMYBL2.1* and *BrLBD38.2* were slightly reduced by 12 °C-treatment in two purple lines, and they were highly expressed and increased in 12 °C-treatment in white head Line94S (Figure 6C,I and Table S1). The other repressors, *BrMYBL2.2*, *BrLBD37.2*, *BrLBD37.3*, *BrLBD38.1* and *BrLBD39.2*, were not sensitive to low temperature in three lines (Figure 6 and Table S1). However, *BrLBD39.2* was highly expressed in three Chinese cabbages at room temperature and down-regulated in purple lines, which suggested that it might be a factor for producing the non-purple appearance of purple lines at room temperature (Figure 6J and Table S1). This implied that up-regulation of *BrMYBL2.1* and *BrLBD38.2* might be one of reasons for promoting non-purple phenotype formation in white head Chinese cabbage.

### 3.7. Classification Scheme of Gene Expression Data and Interaction Networks

**Figure 7:**
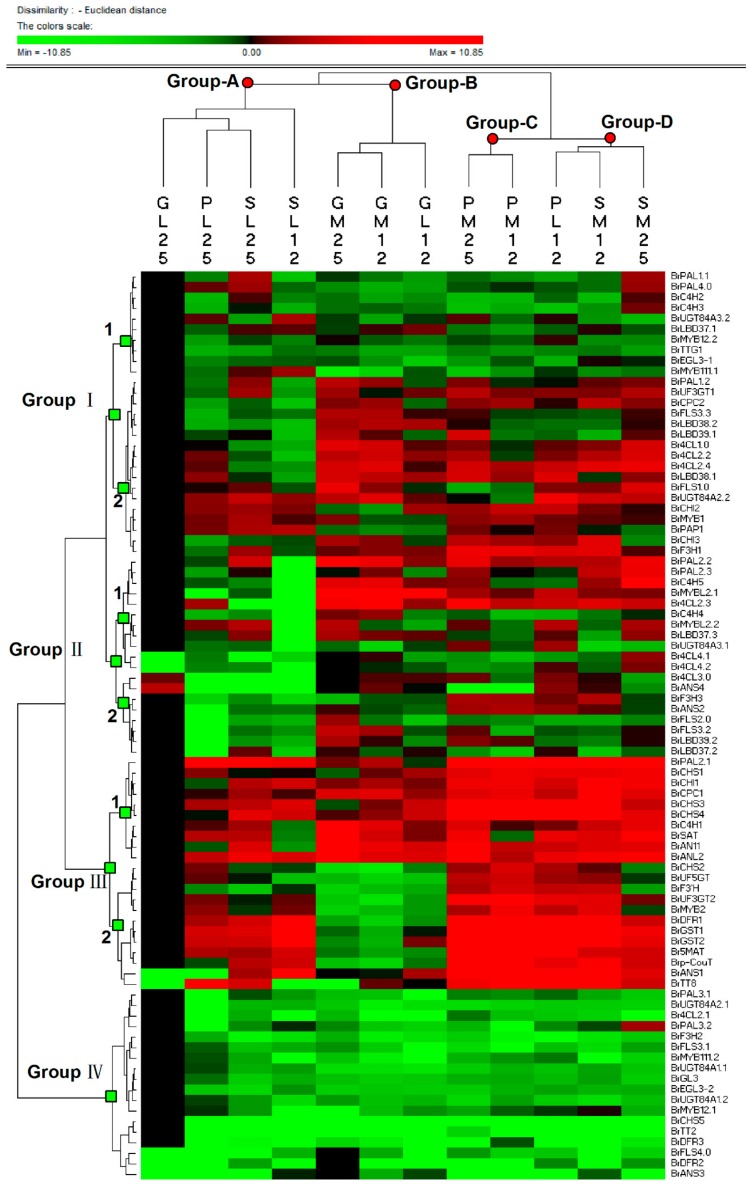
Hierarchical clustering analysis of transcript levels about genes involved in anthocyanin metabolism in seedlings of Chinese cabbages after low temperature treatment. The expression data was log2-normalized, and the clustering analysis was performed using the PermutMatrix graphical software and analyzed with Euclidean distance and Ward’s method. Red boxes indicate higher levels of up-regulation, and green boxes indicate lower levels of down-regulation; the color brightness is directly proportional to the expression ratio. The first capital letters ‘G’, ‘P’, ‘S’ are Line94S17, Line95T2-5, and Line11S91, respectively; the second capital letters ‘L’ and ‘M’ are leaves and midribs, respectively; the third numbers ‘25’ and ‘12’ are 25 °C and 12 °C, respectively.

In hierarchical clustering analysis, columns reflect the different samples, and rows indicate the various genes (Figure 7). Samples with similar gene expression characters are clustered in a branch. In Figure 7, Group-A consisted of leaves, containing 25 °C-treated leaves from three lines, and 12 °C-treated leaves of Line11S; Group-B includes samples only from Line94S, such as 12 °C treated leaves and midribs, and 25 °C treated midribs (Figure 7). Comparatively, Group-C only contains two midribs of Line95T, whereas two midribs of Line11S and 12 °C-treated leaves of Line95T are in Group-D. Expressions about anthocyanin-related genes in three Chinese cabbages elucidated that there was a significant change between Line94S and purple lines after low temperature treatment. Genes with similar trends between Line11S and Line95T implied that the seedling midrib part of Line11S inherited the characteristic of flavonoid biosynthesis from the purple trait donor, whereas the soft leaf proportion inherited the corresponding expression characteristic from white head Chinese cabbage.

In row classification, genes were able to be classified into four groups. Genes scarcely expressed in samples are clustered in Group-IV, including PMPGs *BrPAL3.1*, *BrPAL3.2*, and *Br4CL2.1*, EBGs *BrCHS5*, *BrF3H2*, *BrFLS3.1*, and *BrFLS4.0*, LBGs *BrDFR2*, *BrDFR3*, *BrANS3*, *BrUGT84A1.1*, *BrUGT84A1.2*, and *BrUGT84A2.1*, and positive regulators *BrMYB12.1*, *BrMYB111.2*, *BrGL3*, *BrEGL3-2*, and *BrTT2* (Figure 7). Group-I, Group-II, and Group-III, with different degrees of up-regulation, were divided into two subgroups, respectively. Genes with high expressions in purple lines were in Group-III.1, including *BrPAL2.1*, *BrC4H1*, *BrCHS1*, *BrCHS3*, *BrCHS4*, *BrCHI1*, *BrSAT*, *BrCPC1*, *BrAN11*, and *BrANL2*; EBGs *BrCHS3*, *BrCHS4*, and *BrCHI1* moderately positively responded to low temperature in purple lines (Figure 7). Comparatively, genes in Group-III.2 were significantly induced by low temperature in purple lines and were undetectable in white head Chinese cabbage seedlings, listed as follows: EBGs *BrCHS2* and *BrF3’H*, LBGs *BrDFR1*, *BrANS1*, *BrUF3GT2*, *BrUF5GT*, *Br5MAT*, *Brp-Cout*, *BrGST1*, and *BrGST2*, a positive R2R3 regulatory gene *BrMYB2*, and a bHLH gene *BrTT8* (Figure 7). PMPGs, EBGs, and regulatory genes in Group-I.1 were nearly down-regulated except for 25 °C-treated midribs and leaves of Line11S; genes in Group-I.2 were highly expressed in midribs of three lines, containing PMPGs, EBGs, and several regulator genes (Figure 7). Group-II consisted of genes scarcely expressed in non-purple seedling leaves of three lines, but they were highly expressed in the white line. For example, negative regulators *BrMYBL2.1*, *BrMYBL2.2*, and a series of PMPGs were in Group-II.1 (Figure 7). 

**Figure 8:**
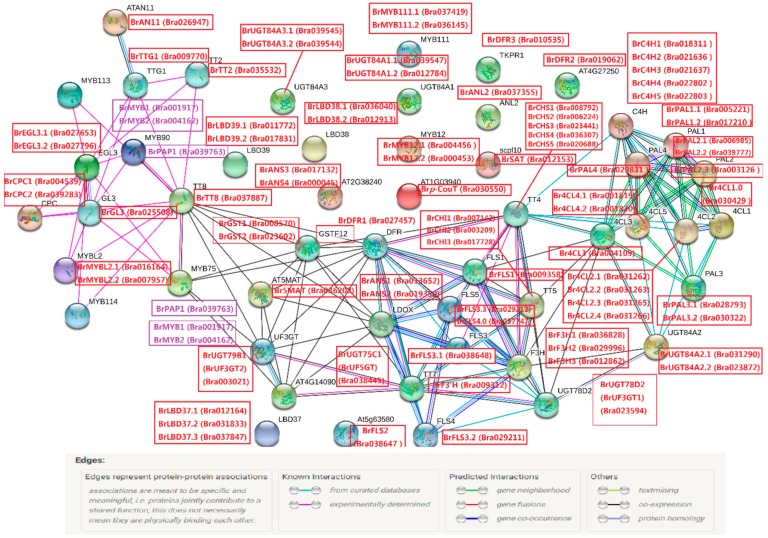
The putative interaction networks of anthocyanin biosynthesis genes in purple head Chinese cabbage. The homologous genes from Chinese cabbage and *Arabidopsis* are in red and black, respectively. Three R2R3-MYB TFs ‘BrMYB1’, ‘BrMYB2’, and ‘BrPAP1’ are highly homologous to MYB90 (AtPAP2, AT1G66390) and MYB75 (AtPAP1, AT1G56650).

To predict the protein interactions of anthocyanin biosynthesis genes, a putative interaction network was constructed in STRING software according to the orthologous proteins in *Arabidopsis* (Figure 8). A total of 86 anthocyanin biosynthesis proteins associated with 51 known *Arabidopsis* proteins were involved in the network. A central bridge node ‘AtTT8’ (AT4G09820, coding a bHLH TF) was associated with most regulatory proteins in the pathway, including ten regulatory TFs and six late biosynthesis members (AtDFR, AtLDOX, AtUF3GT, At5MAT, and AtGST) (Figure 8). The positive TFs were R2R3-MYB factors ‘AtPAP1’, ‘AtPAP2’, ‘AtMYB113’, ‘AtMYB114’ and ‘AtTT2’, bHLH TFs ‘AtGL3’ and ‘AtEGL3’, and a WD40 TF ‘AtTTG’; the negative TFs were R3-MYB TFs ‘AtCPC’ and ‘AtMYBL2’ (Figure 8). The protein–protein associations revealed that AtTT8 was most likely co-expressed with AtDFR, AtLDOX, AtUF3GT, At5MAT, and AtGST (Figure 8). Proteins of PMPGs with multiple copies showed gene neighborhood associations and some of them displayed a gene co-occurrence characteristic, such as *AtPAL1*, *AtPAL2*, and *AtPAL4*; however, proteins of EBGs (AtCHS, AtCHI, AtF3H, and AtF3’H) had co-occurrence and co-expression characteristics, which showed similarity in LBG proteins ‘AtLDOX’ and ‘AtDFR’ (Figure 8). Interestingly, associations among several proteins of decorating LBGs were only of co-expression, such as UF3GT, AT4G914090, and At5MAT (Figure 8). Taken together the expression patterns, analysis of interaction networks suggested that BrMYB2 could strongly interact with BrTT8 in putative co-expression to regulate downstream genes, such as *BrDFR1*, *BrANS1*, *BrUF3GT2*, *Br5MAT*, and *BrGSTs*, enhancing the understanding of metabolism of anthocyanin biosynthesis in purple head Chinese cabbage under the low temperature condition (Figure 8 and Table S2).

## 4. Discussion

Cold stress stimulates anthocyanin biosynthesis by up-regulating the expression of TF genes, and anthocyanin biosynthetic genes have been well documented in recent years [1,21,23,24,25,31,48]. For example, MdMYBA specifically combines to the *ANS* promoter and activates anthocyanin production under low temperature conditions in apple skin [49], whereas MdbHLH3 binds to the promoters of *MdDFR* and *MdUFGT*, accompanied with a regulatory *MdMYB1* gene to activate their expressions [50]. In addition, a NAC TF is selectively induced by cold storage in blood oranges rather than common oranges, and anthocyanins accumulate in vesicles with up-regulation of *PAL*, *CHS*, *DFR*, and *UFGT* [51]. MdSIZ1 (a small ubiquitin-like modifier E3 ligase) promotes anthocyanin biosynthesis by sumoylating MdMYB1 under low temperature conditions (17 °C) in apple [52]. Anthocyanin accumulation in *Arabidopsis* seedlings is stimulated by low temperature treatment with up-regulation of *AtCHS*, *AtCHI*, and *AtF3H*, and HY5 and HYH regulators play important roles in this process [33]. In purple kale, up-regulation of *BoPAP2* and anthocyanin biosynthetic genes was induced by low temperature during anthocyanin accumulation, and the up-regulation of BoPAP1 accompanied by the down-regulation of BoMYB113 and BoMYB114 may be a unique mechanism to regulate anthocyanin accumulation in purple kale under low temperature stress [31]. However, many details of the underlying regulatory networks remain to be elucidated in *Brassica* crops in cold conditions. Here, we performed a transcriptional analysis of 86 anthotyanin biosynthesis genes in leaves and midribs from Chinese cabbage seedlings grown at room temperature (25 °C) and low temperature (12 °C) to identify genes involved in the mechanism of anthocyanin accumulation.

In this report, we demonstrated that anthocyanin biosynthesis in Chinese cabbage seedling is greatly raised by low temperature, producing purple color development after 15 days of a certain extent of cold treatment (such as 12 °C in our results). Low temperature promoted high expression of positive regulator genes *BrMYB2* and *BrTT8*, as well as a series of biosynthesis genes in the anthocyanin pathway in purple head Chinese cabbage and its purple trait donor, accompanied with large accumulations of anthocyanins in one to two cell layers under the midrib epidermis. These results were similar to the report about Japanese parsley, which strongly responded to low temperature stress with four-fold higher anthocyanin accumulation in parsley plants at 12 °C [53]. In addition, low temperature at 15 °C also promoted high content of anthocyanins in red 35S:*PAP1 Arabidopsis* [54]. The 16 °C low temperature and 30 °C high temperature promoted and inhibited anthocyanin accumulation in crabapple leaves, respectively [26]. Thus, we speculated that the low temperature during the head formation process might increase anthocyanin accumulation in both purple head Chinese cabbage and its purple parent.

Apart from low temperature to anthocyanin accumulation in plants, corresponding gene expression patterns are also important to better understand the metabolism of anthocyanin biosynthesis. In apple, Xie et al. found that the cold-induced bHLH TF gene ‘*MdbHLH3*’ bound to the promoters of the LBGs ‘*MdDFR*’ and ‘*MdUFGT*’, and the regulatory gene ‘*MdMYB1*’ activates their expression, which demonstrates that the molecular coloration mechanism of the apple is low temperature-induced [50]. The functions of anthocyanin biosynthesis genes *VvF3H*, *VvPAL*, *VvCHS3*, *VvCHS2*, and *VvLDOX* in grapevine are conservative and sensitive to low temperature [24]. Similarly, *PAL*, *CHS*, *DFR*, and *UFGT* were strongly induced during low temperature in red orange [28]; expression of *CHS*, *ANS*, and *UFGT* was enhanced along with anthocyanin production in the skin of apple fruit under low temperature treatment [30]. In crabapple leaves, biosynthetic genes *McCHS*, *McF3H*, and *McDFR*, and regulatory gene *McMYB10* were involved in the low temperature-induced anthocyanin accumulation [26,55]. It was found that these reports mainly concentrated on the study of the main R2R3-MYBs and several structural genes under low temperature, but the other copies of these genes were scarcely mentioned. Multiple copies of anthocyanin biosynthesis genes were produced during whole genome duplication evolution of *Brassica* from *Arabidopsis*; more PMGs, EBGs, and negative regulatory genes were retained than LBGs and positive regulatory genes [35].

In our reports, nearly all the PMPGs were significantly down-regulated under low temperature, and only *BrPAL2.1* was highly expressed in purple lines, though it was also reduced at 12 °C treatment, implying *BrPAL2.1* was involved in the biosynthesis pathway to supply substrates for following actions. Comparatively, investigated EBGs and corresponding MYB regulators were moderately up-regulated by several times, including *BrCHS2*, *BrCHS3*, *BrCHS4*, *BrCHI1*, *BrCHI2*, *BrCHI3*, *BrF3H1*, *BrF3H3*, *BrF3’H*, and *BrMYBs*. For example, *BrMYB12.1* related to the activation of EBGs in response to low temperature in midribs; however, *BrMYB111.1* and *BrMYB111.2* showed a small increase to low temperature in leaves of Line11S and Line95T, respectively. Notably, the low temperature strongly promoted expressions of LBGs such as *BrDFR1*, *BrANS1*, *BrUGTs* (*BrUF3GT2*, *BrUF5GT*, and *BrUGT84A2.2*), *BrGSTs* (*BrGST1* and *BrGST2*), and several acyltransferase genes (*Br5MAT* and *Brp-Cout*). Hence, we further verified that PMPGs involved in initial phenylpropanoid metabolic pathway and LBGs involved in late anthocyanin biosynthesis are much more sensitive than EBGs to low temperature treatment. Based on extensive studies of plants, an increasing number of evidence demonstrated that anthocyanin biosynthesis is usually activated by MBW complex in transcriptional control of LBGs, and loss-of-function mutant in these genes often lead to the colorless phenotype [4,56]. In addition, the mechanism of different key MYB factors controlling anthocyanin biosynthesis may be independent or synergetic to regulate anthocyanin accumulation in a plant, and members of them show a tissue-specific character. For instance, MYB genes *PhAN2*, *PhAN4*, *PhPHZ*, and *PhDPL* determine the petunia flower color [57,58,59]. PhAN2 determines the red coloration in corolla, and PhAN4 interacts with bHLH TF ‘PhAN1’ to produce red color in anthers and corolla tubes [57]. However, *PhDPL* affects the red corolla tube color, and *PhPHZ* manages blushing of the flower bud [58,59]. In snapdragon, AmROSEA1 activates *F3H*, *F3’H*, *DFR*, *FLS*, and *UFGT* to produce strong red coloration in both epidermal layers of the corolla, whereas AmROSEA2 positively regulates *F3’H* to form weak pigmentation in the inner epidermal layer of the corolla. AmVENOSA up-regulates *CHI*, *F3H*, *F3’H*, *FLS*, *ANS*, and *UFGT* to govern the venation coloration in petals [60]. It appears that *BrMYB2* is a key gene to activate the up-regulation of structural genes under low temperature conditions in purple head Chinese cabbage and its purple trait donor. In addition, we also investigated two members, namely *BrAN11* and *BrANL2* (homology to *AtAN11* and *AtANL2* in *Arabidopsis*, respectively), which were thought to be reacting in anthocyanin production. The *AtANL2* encodes a homeodomain protein of the HD-GLABRA2 group, involved in the accumulation of anthocyanin and root development [61]; the *BrAN11* encodes a LIGHT-REGULATED WD1 involved in the anthocyanin-containing compound biosynthetic process (Annotations in TAIR). However, these two genes were highly expressed in the white line and reduced in the low temperature condition in three lines (Figure S1), indicating that *BrAN11* and *BrANL2* might not be responsible to anthocyanin accumulation under cold conditions in purple head Chinese cabbage.

**Figure 9:**
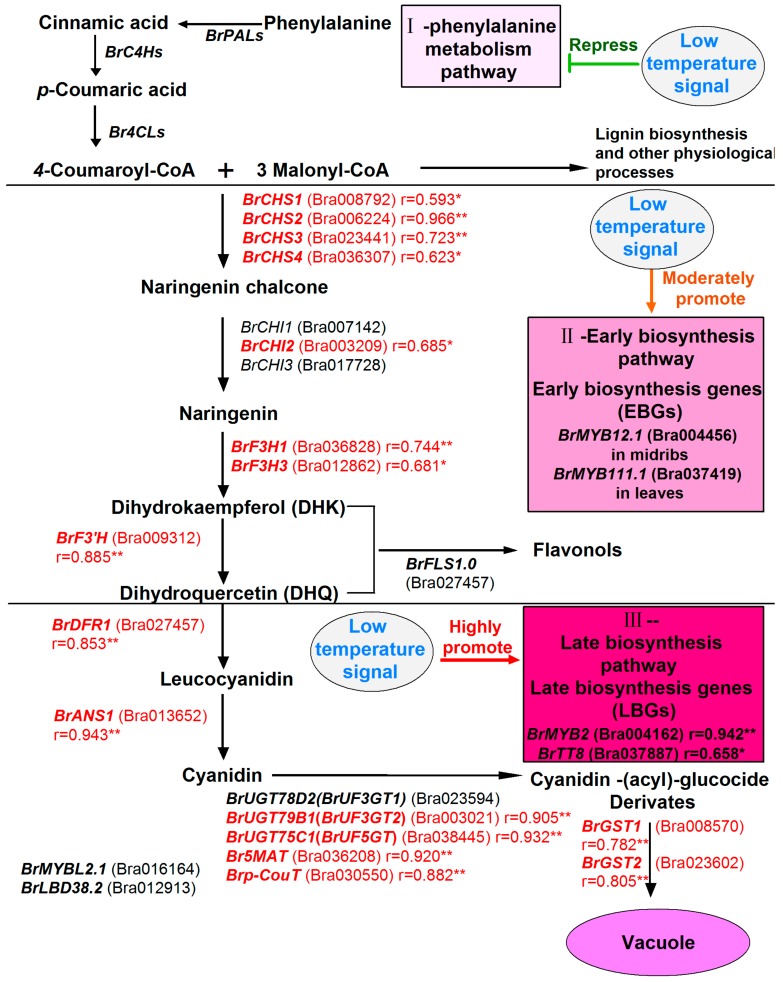
Potential biosynthetic pathway of anthocyanin under the low temperature condition in seedlings of purple head Chinese cabbage. The relative coefficient r with “*” and “**” indicates significant and highly significant correlations between gene expression and total anthocyanin content at the level of 0.05 and 0.01, respectively.

As previously mentioned, negative regulators play important roles in the flavonoid biosynthesis pathway. The single repeat R3-MYB TFs ‘*AtMYBL2*’ and ‘*AtCPC*’, and LBD members ‘*AtLBD37*’, ‘*AtLBD38*’, and ‘*AtLBD39*’, are identified negative regulatory genes of anthocyanin biosynthesis in *Arabidopsis* [17,18,19]. The inhibition of *AtMYBL2* on anthocyanin biosynthesis is usually by repressing the structural genes or by directly inhibiting the MBW complex activity to biosynthesis genes, and it also represses the expression level of MYB and bHLH genes such as *AtTT8*, *AtPAP1*, and *AtPAP2* [12]. A recent report about purple cabbage (*B. oleracea* var. *capitata* F. *rubra*) disclosed that the function loss created by coding sequence or promoter of *BoMYBL2.1* is responsible for producing purple cabbage. *BoMYBL2.1* showed a reverse expression pattern to total anthocyanin content [62]. Overexpression of *MdMYBL2* in red-fleshed apple callus reduced anthocyanin contents, and it strongly repressed the expression of *MdDFR*, *MdUFGT*, *MdMYB10*, and *MdbHLH3* by interacting with MdbHLH3 [63]. *AtLBD37*, *AtLBD38*, and *AtLBD39* are able to suppress the key regulators *AtPAP1* and *AtPAP2* of anthocyanin biosynthesis in *Arabidopsis* [19]. Overexpression of *MdLBD13 in Arabidopsis* repressed anthocyanin accumulation and expression of *AtPAP1* to down-regulate *AtCHS*, *AtCHI*, *AtDFR1*, and *AtUFGT*, and it also reduced the nitrate assimilation [64]. These members had multiple copies in the *Brassica* genome and some of them actively responded to low temperature signals, such as *BrMYBL2.1* and *BrLBD38.2*. *BrMYBL2.1* and *BrLBD38.2* were highly expressed at room temperature and increased in Line94S after 12 °C treatment, which implied that *BrMYBL2.1* and *BrLBD38.2* might positively affect the non-purple phenotype in white head Chinese cabbage. Comparatively, *BrLBD39.2* was highly expressed in all Chinese cabbages at room temperature, and significantly down-regulated in two purple lines, which implied that it might be a factor in lightening the purple appearance of purple lines at room temperature. Taken together, a network about the expression patterns of genes to better understand anthocyanin biosynthesis in purple Chinese cabbage seedlings under low temperature conditions was constructed (Figure 9). The initial phenylpropanoid metabolic pathway and early biosynthesis pathway occurs in all Chinese cabbages at room temperature, but the phenylpropanoid metabolic pathway is nearly repressed; low temperature induced moderate up-regulation of several EBGs and generated up-regulation of LBGs and related regulators in seedling of purple lines. These implied that initial phenylpropanoid metabolic pathway and early biosynthesis process might maintain normal physiological metabolism, and the PMPGs might be more redundant for normal physiological metabolism in Chinese cabbages. Though *BrPAL2.1* was down-regulated after cold induction, it still remained relatively high expression in purple samples under cold condition. Perhaps the up-regulation of *BrPAL2.1* produces more precursors for following biosynthesis under low temperature conditions.

## 5. Conclusions

In conclusion, low temperature induced a R2R3-MYB regulator *BrMYB2* and a bHLH regulator *BrTT8* to activate the late anthocyanin biosynthesis in purple Chinese cabbages, but the induction of two negative regulators *BrMYBL2.1* and *BrLBD38.2* in white head Chinese cabbage might promote the formation of a common phenotype. In addition, the significant up-regulation of *BrMYB2* and *BrTT8*, accompanied by the slight up-regulation of *BrPAP1* and an obvious down-regulation of *BrMYB1* and *BrMYBL2.1*, might disclose a new unique mechanism for regulating anthocyanin biosynthesis in purple head Chinese cabbage seedlings under low temperature stress. As a future target, we will focus on the function of the MYB and bHLH TFs, together with interactions of other anthocyanin biosynthesis factors.

## Supplementary Materials

The following are available online at https://www.mdpi.com/2073-4425/11/1/81/s1, Figure S1: Expression of *BrAN11* and *BrANL2* in the seedlings of three Chinese cabbages after low temperature treatment. The leaves of Line94S at 25 °C were served as the control; 28-day seedlings were treated at 12 °C for 15 days for the experiment. Values are presented as means ± SD (*n* = 3). The different letters above each column are significantly different at *p* < 0.05 by Duncan’s test; Figure S2: The putative interaction networks of regulatory genes involved in anthocyanin biosynthesis in purple head Chinese cabbage. The homologous genes from Chinese cabbage and *Arabidopsis* are in red and black, respectively. Three R2R3-MYB TFs ‘BrMYB1’, ‘BrMYB2’, and ‘BrPAP1’ are highly homologous to MYB90 (AtPAP2, AT1G66390) and MYB75 (AtPAP1, AT1G56650); Table S1; Relative expressions of anthocyanin biosynthesis in seedlings of Chinese cabbage under 25 °C and 12 °C conditions. Plants of three lines were grown under a 12-h light photoperiod at 25 °C, 125 mmol m^−2^s^−1^; 28-day seedlings were kept at 12 °C for 15 days, and the seedlings that stayed at 25 °C with same light condition were treated as the control. The means are values ± SD. The different letters in each line are significantly different at *p* < 0.05 by Duncan’s test; Table S2: Genes involved in anthocyanin biosynthesis in *B. rapa*. The information was collected from the *Brassica* database. *At*, *Arabidopsis*; *Br*, *Brassica*; Table S3; Primers for the qRT-PCR analysis in Chinese Cabbages. a: Sequence length was acquired according to the BRAD database.

## References

[1] Bi H.T., Guo M.L., Wang J.W., Qu Y., Du W.L., Zhang K.M. (2018). Transcriptome analysis reveals anthocyanin acts as a protectant in *Begonia semperflorens* under low temperature. Acta Physiol. Plant..

[2] Wiczkowski W., Topolska J., Honke J. (2014). Anthocyanins profile and antioxidant capacity of red cabbages are influenced by genotype and vegetation period. J. Funct. Foods.

[3] Song T., Li K., Wu T., Wang Y., Zhang X., Xu X., Yao Y., Han Z. (2019). Identification of new regulators through transcriptome analysis that regulate anthocyanin biosynthesis in apple leaves at low temperatures. PLoS ONE.

[4] Naing A.H., Kim C.K. (2018). Roles of R2R3-MYB transcription factors in transcriptional regulation of anthocyanin biosynthesis in horticultural plants. Plant Mol. Biol..

[5] Macdonald M., D’Cunha G. (2007). A modern view of phenylalanine ammonia lyase. Biochem. Cell Biol..

[6] Li Y., Kim J.I., Pysh L., Chapple C. (2015). Four isoforms of *Arabidopsis thaliana* 4-coumarate: CoA ligase (4CL) have overlapping yet distinct roles in phenylpropanoid metabolism. Plant Physiol..

[7] Zhang X.P., Xu Z.D., Yu X.Y., Zhao L.Y., Zhao M.Y., Han X., Qi S. (2019). Identification of two novel R2R3-MYB transcription factors, PsMYB114L and PsMYB12L, related to anthocyanin biosynthesis in *Paeonia suffruticosa*. Int. J. Mol. Sci..

[8] Sun W., Meng X., Liang L., Jiang W., Huang Y., He J., Hu H., Almqvist J., Gao X., Wang L. (2015). Molecular and Biochemical Analysis of Chalcone Synthase from Freesia hybrid in Flavonoid Biosynthetic Pathway. PLoS ONE.

[9] Pelletier M.K., Shirley B.W. (1997). Characterization of flavonol synthase and leucoanthocyanidin dioxygenase genes in Arabidopsis. Further evidence for differential regulation of “early” and “late” genes. Plant Physiol..

[10] Albert N.W., Davies K.M., Lewis D.H., Huaibi Z., Mirco M., Cyril B., Boase M.R., Hanh N., Jameson P.E., Schwinn K.E. (2014). A conserved network of transcriptional activators and repressors regulates anthocyanin pigmentation in eudicots. Plant Cell.

[11] Liu Y.J., Hua H., Jiang X.L., Wang P.Q., Tao X. (2018). A WD40 repeat protein from *Camellia sinensis* regulates anthocyanin and proanthocyanidin accumulation through the formation of MYB–bHLH–WD40 ternary complexes. Int. J. Mol. Sci..

[12] Petroni K., Tonelli C. (2011). Recent advances on the regulation of anthocyanin synthesis in reproductive organs. Plant Sci..

[13] Stracke R., Ishihara H., Huep G., Barsch A., Mehrtens F., Niehaus K., Weisshaar B. (2007). Differential regulation of closely related R2R3-MYB transcription factors controls flavonol accumulation in different parts of the Arabidopsis thaliana seedling. Plant J..

[14] Fu Z.Z., Wang L.M., Shang H.Q., Dong X.Y., Jiang H., Zhang J., Wang H.J., Li Y.M., Yuan X., Meng S.Y. (2019). An R3-MYB gene of *Phalaenopsis*, *MYBx1*, represses anthocyanin accumulation. Plant Growth Regul..

[15] Baudry A., Heim M.A., Dubreucq B., Caboche M., Weisshaar B., Lepiniec L. (2004). TT2, TT8, and TTG1 synergistically specify the expression of *BANYULS* and proanthocyanidin biosynthesis in *Arabidopsis thaliana*. Plant J..

[16] Dubos C., Le Gourrierec J., Baudry A., Huep G., Lanet E., Debeaujon I., Routaboul J.M., Alboresi A., Weisshaar B., Lepiniec L. (2008). MYBL2 is a new regulator of flavonoid biosynthesis in *Arabidopsis thaliana*. Plant J..

[17] Kyoko M., Yoshimi U., Masaru O.T. (2010). AtMYBL2, a protein with a single MYB domain, acts as a negative regulator of anthocyanin biosynthesis in *Arabidopsis*. Plant J..

[18] Zhu H.F., Fitzsimmons K., Khandelwal A., Kranz R.G. (2009). CPC, a single-repeat R3 MYB, is a negative regulator of anthocyanin biosynthesis in *Arabidopsis*. Mol. Plant.

[19] Rubin G., Tohge T., Matsuda F., Saito K., Scheible W.R. (2009). Members of the LBD family of transcription factors repress anthocyanin synthesis and affect additional nitrogen responses in Arabidopsis. Plant Cell Online.

[20] Page M., Sultana N., Paszkiewicz K., Florance H., Smirnoff N. (2012). The influence of ascorbate on anthocyanin accumulation during high light acclimation in Arabidopsis thaliana: Further evidence for redox control of anthocyanin synthesis. Plant Cell Environ..

[21] Fang H.C., Dong Y.H., Yue X.X., Hu J.F., Jiang S.H., Xu H.F., Wang Y.C., Su M.Y., Zhang J., Zhang Z.Y. (2019). The B-box zinc finger protein MdBBX20 integrates anthocyanin accumulation in response to ultraviolet radiation and low temperature. Plant Cell Environ..

[22] Landi M., Pardossi A., Remorini D., Guidi L. (2013). Antioxidant and photosynthetic response of a purple-leaved and a green-leaved cultivar of sweet basil (*Ocimum basilicum*) to boron excess. Environ. Exp. Bot..

[23] Zhang Y.T., Liu Y., Hu W.J., Sun B., Chen Q., Tang H.R. (2018). Anthocyanin accumulation and related gene expression affected by low temperature during strawberry coloration. Acta Physiol. Plant..

[24] Zhang C., Jia H.F., Wu W.M., Wang X.C., Fang J.G., Wang C. (2015). Functional conservation analysis and expression modes of grape anthocyanin synthesis genes responsive to low temperature stress. Gene.

[25] Gao-Takai M., Katayama-Ikegami A., Matsuda K., Shindo H., Uemae S., Oyaizu M. (2019). A low temperature promotes anthocyanin biosynthesis but does not accelerate endogenous abscisic acid accumulation in red-skinned grapes. Plant Sci..

[26] Tian J., Han Z.Y., Zhang L.R., Song T.T., Zhang J., Li J.Y., Yao Y. (2015). Induction of anthocyanin accumulation in crabapple (*Malus cv*.) leaves by low temperatures. Hortsci. Publ. Am. Soc. Hortic. Sci..

[27] Leng P., Qi J.X. (2003). Effect of anthocyanin on David peach (*Prunus davidiana* Franch) under low temperature stress. Sci. Hortic..

[28] Lo Piero A.R., Puglisi I., Rapisarda P., Petrone G. (2005). Anthocyanins accumulation and related gene expression in red orange fruit induced by low temperature storage. J. Agric. Food Chem..

[29] Wang N., Qu C., Jiang S., Chen Z., Xu H., Fang H., Su M., Zhang J., Wang Y., Liu W. (2018). The proanthocyanidin-specific transcription factor MdMYBPA1 initiates anthocyanin synthesis under low-temperature conditions in red-fleshed apples. Plant J..

[30] Ubi B.E., Honda C., Bessho H., Kondo S., Wada M., Kobayashi S., Moriguchi T. (2006). Expression analysis of anthocyanin biosynthetic genes in apple skin: Effect of UV-B and temperature. Plant Sci..

[31] Zhang B., Hu Z.L., Zhang Y.J., Li Y.L., Zhou S., Chen G.P. (2012). A putative functional MYB transcription factor induced by low temperature regulates anthocyanin biosynthesis in purple kale (*Brassica Oleracea* var. *acephala* f. *tricolor*). Plant Cell Rep..

[32] Landi M., Tattini M., Gould K.S. (2015). Multiple functional roles of anthocyanins in plant-environment interactions. Environ. Exp. Bot..

[33] Zhang Y.Q., Zheng S., Liu Z.J., Wang L.G., Bi Y.R. (2011). Both HY5 and HYH are necessary regulators for low temperature-induced anthocyanin accumulation in *Arabidopsis* seedlings. J. Plant Physiol..

[34] Sun B.M., Zhu Z.S., Cao P.R., Chen H., Chen C.M., Zhou X., Mao Y.H., Lei J.J., Jiang Y.P., Meng W. (2016). Purple foliage coloration in tea (*Camellia sinensis* L.) arises from activation of the R2R3-MYB transcription factor CsAN1. Sci. Rep..

[35] Guo N., Cheng F., Wu J., Liu B., Zheng S.N., Liang J.L., Wang X.W. (2014). Anthocyanin biosynthetic genes in *Brassica rapa*. BMC Genom..

[36] Ahmed N.U., Park J.I., Jung H.J., Hur Y., Nou I.S. (2015). Anthocyanin biosynthesis for cold and freezing stress tolerance and desirable color in *Brassica rapa*. Funct. Integr. Genom..

[37] Ahmed N.U., Park J.I., Jung H.J., Yang T.J., Hur Y., Nou I.S. (2014). Characterization of dihydroflavonol 4-reductase (DFR) genes and their association with cold and freezing stress in *Brassica rapa*. Gene.

[38] Lux A., Morita S., Abe J., Ito K. (2005). An improved method for clearing and staining free-hand sections and whole-mount samples. Ann. Bot..

[39] He Q., Zhang Z.F., Zhang L.G. (2016). Anthocyanin Accumulation, Antioxidant Ability and Stability, and a Transcriptional Analysis of Anthocyanin Biosynthesis in Purple Heading Chinese Cabbage (*Brassica rapa* L. ssp. *pekinensis*). J. Agric. Food Chem..

[40] Giusti M.M., Wrolstad R.E. (2001). Characterization and Measurement of Anthocyanins by UV-Visible Spectroscopy. Current Protocols in Food Analytical Chemistry.

[41] Livak K.J., Schmittgen T.D. (2001). Analysis of relative gene expression data using real-time quantitative PCR and the 2-△△CT method. Methods.

[42] Caraux G., Pinloche S. (2005). PermutMatrix: A graphical environment to arrange gene expression profiles in optimal linear order. Bioinformatics.

[43] Szklarczyk D., Morris J.H., Cook H., Kuhn M., Wyder S., Simonovic M., Santos A., Doncheva N.T., Roth A., Bork P. (2017). The STRING database in 2017: Quality-controlled protein–protein association networks, made broadly accessible. Nucleic Acids Res..

[44] Gou J.Y., Felippes F.F., Liu C.J., Weigel D., Wang J.W. (2011). Negative regulation of anthocyanin biosynthesis in Arabidopsis by a miR156-targeted SPL transcription factor. Plant Cell Online.

[45] Fraser C.M., Thompson M.G., Shirley A.M., Ralph J., Schoenherr J.A., Sinlapadech T., Hall M.C., Chapple C. (2007). Related Arabidopsis serine carboxypeptidase-like sinapoylglucose acyltransferases display distinct but overlapping substrate specificities. Plant Physiol..

[46] Keiko Y.S., Fukushima A., Nakabayashi R., Hanada K., Matsuda F., Sugawara S., Inoue E., Kuromori T., Ito T., Shinozaki K. (2012). Two glycosyltransferases involved in anthocyanin modification delineated by transcriptome independent component analysis in *Arabidopsis thaliana*. Plant J..

[47] Meißner D., Albert A., Böttcher C., Strack D., Milkowski C. (2008). The role of UDP-glucose:hydroxycinnamate glucosyltransferases in phenylpropanoid metabolism and the response to UV-B radiation in *Arabidopsis thaliana*. Planta.

[48] Carmona L., Alquezar B., Marques V.V., Pena L. (2017). Anthocyanin biosynthesis and accumulation in blood oranges during postharvest storage at different low temperatures. Food Chem..

[49] Ban Y., Honda C., Hatsuyama Y., Igarashi M., Bessho H., Moriguchi T. (2007). Isolation and functional analysis of a MYB transcription factor gene that is a key regulator for the development of red coloration in apple skin. Plant Cell Physiol..

[50] Xie X.B., Li S., Zhang R.F., Zhao J., Chen Y.C., Zhao Q., Yao Y.X., You C.X., Zhang X.S., Hao Y.J. (2012). The bHLH transcription factor MdbHLH3 promotes anthocyanin accumulation and fruit colouration in response to low temperature in apples. Plant Cell Environ..

[51] Crifo T., Petrone G., Lo Cicero L., Lo Piero A.R. (2012). Short cold storage enhances the anthocyanin contents and level of transcripts related to their biosynthesis in blood oranges. J. Agric. Food Chem..

[52] Zhou L.J., Li Y.Y., Zhang R.F., Zhang C.L., Xie X.B., Zhao C., Hao Y.J. (2017). The small ubiquitin-like modifier E3 ligase MdSIZ1 promotes anthocyanin accumulation by sumoylating MdMYB1 under low-temperature conditions in apple. Plant Cell Environ..

[53] Hajime H., Tomoko F.A., Toshikatsu O., Minoru N., Masahiko S. (2001). Anthocyanin accumulation and related gene expression in Japanese parsley (*Oenanthe stolonifera*, DC.) induced by low temperature. J. Plant Physiol..

[54] Rowan D.D., Cao M., Kui L.W., Cooney J.M., Jensen D.J., Austin P.T., Hunt M.B., Norling C., Hellens R.P., Schaffer R.J. (2009). Environmental regulation of leaf colour in red 35S:*PAP1 Arabidopsis thaliana*. New Phytol..

[55] Tian J., Chen M., Zhang J., Li K., Song T., Zhang X., Yao Y. (2017). Characteristics of dihydroflavonol 4-reductase gene promoters from different leaf colored *Malus* crabapple cultivars. Hortic. Res..

[56] Sakai M., Yamagishi M., Matsuyama K. (2019). Repression of anthocyanin biosynthesis by R3-MYB transcription factors in lily (*Lilium spp*.). Plant Cell Rep..

[57] Albert N.W., Lewis D.H., Zhang H., Irving L.J., Jameson P.E., Davies K.M. (2009). Light-induced vegetative anthocyanin pigmentation in Petunia. J. Exp. Bot..

[58] Spelt C., Quattrocchio F., Mol J.N., Koes R. (2000). *Anthocyanin1* of *petunia* encodes a basic helix-loop-helix protein that directly activates transcription of structural anthocyanin genes. Plant Cell Online.

[59] Albert N.W., Lewis D.H., Huaibi Z., Schwinn K.E., Jameson P.E., Davies K.M. (2011). Members of an R2R3-MYB transcription factor family in Petunia are developmentally and environmentally regulated to control complex floral and vegetative pigmentation patterning. Plant J..

[60] Shang Y.J., Julien V., Steve M., Bailey P.C., Schwinn K.E., Jameson P.E., Martin C.R., Davies K.M. (2011). The molecular basis for venation patterning of pigmentation and its effect on pollinator attraction in flowers of *Antirrhinum*. New Phytol..

[61] Hiroyoshi K., Mutsumi K., Koji G. (2009). Expression analysis of ANTHOCYANINLESS2 gene in *Arabidopsis*. Plant Sci..

[62] Song H., Yi H., Lee M., Han C.T., Lee J., Kim H.R., Park J.I., Nou I.S., Kim S.J., Hur Y. (2018). Purple *Brassica oleracea* var. *capitata* F. *rubra* is due to the loss of BoMYBL2–1 expression. BMC Plant Biol..

[63] Wang Y.C., Sun J.J., Wang N., Xu H.F., Chen X.S. (2019). *MdMYBL2* helps regulate cytokinin-induced anthocyanin biosynthesis in red-fleshed apple (*Malus sieversii* f. niedzwetzkyana) callus. Funct. Plant Biol..

[64] Li H.H., Liu X., An J.P., Hao Y.J., Wang X.F., You C.X. (2017). Cloning and elucidation of the functional role of apple MdLBD13 in anthocyanin biosynthesis and nitrate assimilation. Plant Cell Tissue Organ Cult..

