# Supplementary material for: Low Temperature Promotes Anthocyanin Biosynthesis and Related Gene Expression in the Seedlings of Purple Head Chinese Cabbage (Brassica rapa L.)
Source: Genes (Basel). 2020 Jan 10;11(1):81. doi: 10.3390/genes11010081 (PMC7017278; doi:10.3390/genes11010081)
Supplement: Supplementary file 1 [file genes-11-00081-s001.pdf]

Supplementary Materials for

Low temperature promotes anthocyanin biosynthesis and related gene expression in the seedlings of purple head Chinese cabbage (*Brassica rapa* L.)

Qiong He[1,2], Yanjing Ren[1], Wenbin Zhao[1], Ru Li[1], Lugang Zhang[1, 3]*

[1]State Key Laboratory of Crop Stress Biology for Arid Areas, College of Horticulture, Northwest A&F University, 3 Taicheng Road, Yangling 712100, Shaanxi, People's Republic of China.

[2]College of Life Sciences, Northwest A&F University, 3 Taicheng Road, Yangling 712100, Shaanxi, People's Republic of China.

[3]State Key Laboratory of Vegetable Germplasm Innovation, Tianjin 300192, People's Republic of China.

* E-mail of corresponding author: lugangzh@nwafu.edu.cn; Lugangzh@163.com

Figure S1. Expression of *BrAN11* and *BrANL2* in the seedlings of three Chinese cabbages after low temperature treatment. The leaves of Line94S at 25 °C were served as the control; 28-day seedlings were treated at 12 °C for 15 days for the experiment. Values are presented as means ± SD (n=3). The different letters above each column are significantly different at $p < 0.05$ by Duncan's test.

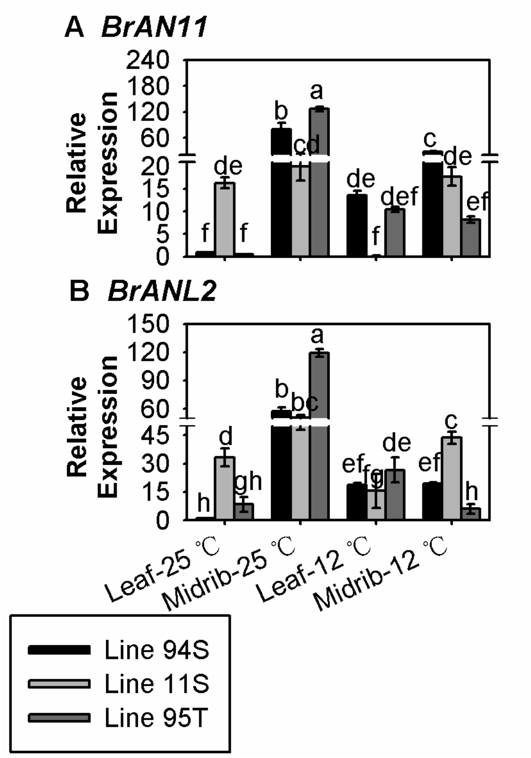

Figure S2. The putative interaction networks of regulatory genes involved in anthocyanin biosynthesis in purple head Chinese cabbage. The homologous genes from Chinese cabbage and *Arabidopsis* are in red and black, respectively. Three R2R3-MYB TFs 'BrMYB1', 'BrMYB2', and 'BrPAP1' are highly homologous to MYB90 (AtPAP2, AT1G66390) and MYB75 (AtPAP1, AT1G56650).

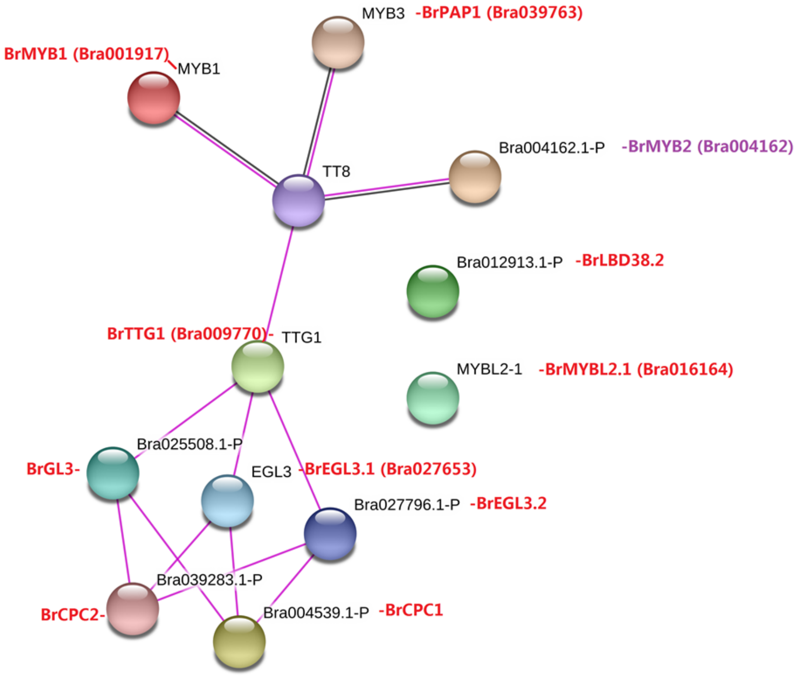

Table S1. Relative expressions of anthocyanin biosynthesis in seedlings of Chinese cabbage under 25 °C and 12 °C conditions. Plants of three lines were grown under a 12 h light photoperiod at 25 °C, 125 mmol $m^{-2}s^{-1}$; 28-day seedlings were kept at 12 °C for 15 days, and the seedlings that stayed at 25 °C with same light condition were treated as the control. The means are values ±SD. The different letters in each line are significantly different at $p < 0.05$ by Duncan's test.

Gene	**BRAD ID**	**Relative expression of detected genes**											
		94S17 Leaf-25 ℃	94S17 Midrib-25 ℃	94S17 Leaf-12 ℃	94S17 Midrib-12 ℃	11S91 Leaf-25 ℃	11S91 Midrib-25 ℃	11S91 Leaf-12 ℃	11S91 Midrib-12 ℃	95T2-5 Leaf-25 ℃	95T2-5 Midrib-25 ℃	95T2-5 Leaf-12 ℃	95T2-5 Midrib-12 ℃
**Structural genes**													
**Biosynthetic genes in phenylpropanoid pathway**													
BrPAL1.1	Bra005221	1.000±0.000b	0.750±0.653b	0.159±0.010b	0.334±0.049b	4.920±0.471a	3.985±1.567a	0.050±0.011b	0.384±0.031b	0.273±0.009b	0.418±0.093b	0.133±0.031b	0.248±0.036b
BrPAL1.2	Bra017210	1.000±0.000de	6.879±0.027a	0.548±0.081e	3.308±0.372b	3.026±0.829b	2.269±1.274bc	0.123±0.015e	1.715±0.250cd	0.356±0.000e	2.355±0.734bc	0.946±0.030de	0.818±0.040de
BrPAL2.1	Bra006985	1.000±0.000f	2.147±0.067f	0.750±0.122f	5.858±1.072f	293.619±67.002b	233.483±19.861c	40.368±22.694ef	139.137±39.243d	740.293±28.284a	348.388±22.846b	84.442±14.900de	56.361±2.011ef
BrPAL2.2	Bra039777	1.000±0.000e	78.352±4.714a	2.821±0.340e	29.307±7.285c	12.320±0.744d	34.214±0.361b	0.000±0.000e	6.123±1.393de	0.648±0.309e	26.778±4.829bc	5.908±1.733de	3.838±1.342e
BrPAL2.3	Bra003126	1.000±0.000de	0.965±0.127de	0.240±0.101de	2.191±0.125cd	0.148±0.024f	3.270±0.903c	0.773±0.209de	1.001±0.883de	1.108±0.053de	30.948±1.538a	0.000±0.000f	8.398±2.213b
BrPAL3.1	Bra028793	1.000±0.000a	0.059±0.000fg	0.000±0.000g	0.051±0.033fg	0.578±0.050b	0.038±0.016fg	0.170±0.047def	0.114±0.036efg	0.000±0.000g	0.287±0.047cd	0.414±0.004c	0.219±0.223de
BrPAL3.2	Bra030322	1.000±0.000b	0.251±0.036b	0.040±0.000b	0.043±0.025b	0.233±0.019b	4.470±1.521a	0.848±0.047b	0.654±0.388b	0.000±0.000b	0.079±0.056b	0.247±0.028b	0.000±0.000b
BrPAL4.0	Bra029831	1.000±0.000c	0.226±0.014cd	0.146±0.026d	0.097±0.007d	3.830±1.134a	3.552±0.106a	0.426±0.088cd	0.393±0.092cd	1.769±0.161b	0.559±0.210cd	0.556±0.034cd	0.835±0.121cd
BrC4H1	Bra018311	1.000±0.000e	57.696±20.707a	2.357±0.381e	20.627±3.432bc	4.231±0.766de	23.540±1.502b	0.334±0.204e	9.709±2.063cde	1.428±0.393e	17.041±0.585bcd	2.137±0.253e	1.383±0.447e
BrC4H2	Bra021636	1.000±0.000b	0.373±0.037cd	0.229±0.025cd	0.128±0.011cd	1.395±0.114a	1.431±0.506a	0.271±0.040cd	0.065±0.010d	0.072±0.000d	0.063±0.010d	0.452±0.078c	0.075±0.011d
BrC4H3	Bra	1.00	0.38	0.33	0.47	0.942	2.046	0.095	0.205	0.095	0.056	0.047	0.110

	021637	0±0.000b	4±0.195cd	1±0.008cd	5±0.007c	±0.448b	±0.090a	±0.017d	±0.022cd	±0.025d	±0.010d	±0.012d	±0.030d
BrC4H4	Bra022802	1.000±0.000c	2.155±0.470b	0.284±0.127de	3.420±0.691a	0.216±0.005e	0.856±0.297cd	0.000±0.000e	0.348±0.104de	0.082±0.059e	0.528±0.019cde	0.081±0.043e	0.066±0.026e
BrC4H5	Bra022803	1.000±0.000d	20.499±2.932c	2.162±0.238d	27.644±2.306b	0.256±0.005d	75.001±8.162a	0.000±0.000d	3.671±0.239d	0.558±0.041d	2.667±0.118d	0.370±0.172d	0.436±0.024d
Br4CL1	Bra030429	1.000±0.000c	20.084±2.201a	1.591±0.160c	12.892±1.540b	0.209±0.007c	14.047±2.434b	0.112±0.026c	2.607±0.392b	1.013±0.116c	2.443±0.155c	1.724±0.297c	0.833±0.071c
Br4CL2.1	Bra031262	1.000±0.000a	0.077±0.017c	0.000±0.000c	0.000±0.000c	0.102±0.001c	0.000±0.000c	0.000±0.000c	0.030±0.005c	0.000±0.000c	0.348±0.300b	0.040±0.000c	0.058±0.009c
Br4CL2.2	Bra031263	1.000±0.000d	7.203±0.100c	2.989±0.722d	11.385±0.325b	0.562±0.110d	15.387±1.636a	0.054±0.007d	6.324±1.805b	1.936±0.401d	5.189±1.174d	2.298±0.210d	0.696±0.121d
Br4CL2.3	Bra031265	1.000±0.000e	42.755±6.139c	3.521±0.785e	79.613±8.448b	0.000±0.000e	10.016±3.577de	0.000±0.000e	21.038±3.177d	4.468±2.648e	102.986±15.516a	10.959±3.497de	8.397±0.391de
Br4CL2.4	Bra031266	1.000±0.000e	14.549±3.343c	1.320±0.082e	20.459±1.831b	0.279±0.077e	28.223±3.919a	0.197±0.001e	22.464±6.055b	1.623±0.266e	13.001±3.552c	9.309±1.575cd	4.755±0.222de
Br4CL3	Bra004109	1.873±0.094ab	1.000±0.000c	1.401±0.607bc	1.453±0.442bc	0.000±0.000d	0.131±0.020d	0.000±0.000d	1.145±0.151c	0.000±0.000d	1.954±0.038a	2.225±0.208a	0.377±0.020d
Br4CL4.1	Bra001819	0.000±0.000d	1.000±0.000b	0.141±0.024cd	1.205±0.103b	0.000±0.000d	3.211±0.424a	0.028±0.000cd	0.405±0.071c	0.317±0.083cd	0.211±0.177cd	0.166±0.041cd	0.055±0.006d
Br4CL4.2	Bra001820	0.000±0.000f	1.000±0.000c	0.549±0.213de	0.855±0.095cd	0.235±0.019ef	2.339±0.660a	0.000±0.000f	0.494±0.121de	0.272±0.031ef	0.175±0.006ef	1.441±0.075b	0.246±0.003ef

Early biosynthetic genes (EBGs)

BrCHS1	Bra008792	1.000±0.000d	0.494±0.005d	4.376±0.300d	1.819±0.147d	0.959±0.142d	40.811±13.768a	1.034±0.361d	27.306±1.541bc	2.665±0.000d	20.629±6.343c	28.567±2.228bc	35.426±4.098ab
BrCHS2	Bra006224	1.000±0.000cde	0.014±0.002e	0.296±0.038e	0.006±0.004e	0.543±0.098de	0.279±0.051e	0.700±0.033de	1.603±0.434cd	2.151±0.132c	3.542±0.881b	4.674±0.314b	15.513±1.621a
BrCHS3	Bra023441	1.000±0.000f	0.616±0.081f	10.011±0.722de	2.209±0.294f	7.478±0.609de	8.887±0.872de	17.185±1.310d	76.024±5.833b	4.851±0.070f	106.611±13.476a	55.943±5.237c	83.512±2.861b
BrCHS4	Bra036307	1.000±0.000f	1.463±0.562f	10.197±1.671f	2.992±0.739f	22.996±3.442e	22.857±2.453e	11.630±2.314ef	116.414±3.865c	0.967±0.213f	429.465±13.403a	96.745±8.043d	234.773±10.316b
BrCHS5	Bra020688	1.000±0.000a	0.000±0.000b	0.000±0.000b	0.000±0.000b	0.000±0.000b	0.000±0.000b	0.000±0.000b	0.000±0.000b	0.000±0.000b	0.000±0.000b	0.000±0.000b	0.000±0.000b
BrCHI1	Bra007	1.000±0.	2.377±0.	2.176±0.	4.762±0.	6.214±1.41	37.950±1.1	12.901±2.6	79.067±3.1	0.578±0.06	31.306±3.0	13.234±0.9	37.590±3.3

	142	000f	263ef	423ef	477ef	3e	47b	20d	35a	9f	00c	51d	86b
BrCHI2	Bra003209	1.000±0.000cde	0.436±0.271de	4.067±1.132b	0.164±0.025e	3.882±1.228b	1.166±0.953cde	2.491±0.135bcd	2.058±0.521bcde	2.884±1.695bc	3.472±0.951b	8.927±0.663a	9.036±1.934a
BrCHI3	Bra017728	1.000±0.000f	4.837±0.107c	0.674±0.265f	2.657±0.272e	0.531±0.269f	0.260±0.092f	0.633±0.192f	19.182±0.686a	0.131±0.000f	6.917±0.993b	3.149±0.594de	4.080±0.625cd
BrF3H1	Bra036828	1.000±0.000d	1.808±1.904d	2.329±0.144d	2.647±0.377d	3.820±2.097d	1.448±1.091d	0.587±0.163d	22.646±1.025bc	0.384±0.251d	33.833±14.266a	17.425±2.314c	27.746±0.843ab
BrF3H2	Bra029996	1.000±0.000a	0.097±0.012b	0.014±0.005d	0.005±0.004d	0.003±0.002d	0.039±0.011c	0.014±0.010d	0.010±0.007d	0.106±0.019b	0.034±0.012c	0.000±0.000d	0.003±0.002d
BrF3H3	Bra012862	1.000±0.000bc	0.067±0.017bc	0.571±0.211bc	0.560±0.086bc	0.219±0.068bc	0.705±0.175bc	0.024±0.011c	5.692±0.760a	0.029±0.020c	4.987±2.563a	1.989±0.394b	4.276±0.852a
BrF3'H	Bra009312	1.000±0.000e	0.027±0.005f	0.111±0.037f	0.062±0.019f	0.043±0.031f	0.153±0.017f	0.801±0.269ef	9.177±0.713b	0.251±0.184f	5.306±0.154d	7.955±0.611c	13.082±0.625a
BrFLS1	Bra009358	1.000±0.000c	28.604±12.937a	0.834±0.088c	2.816±0.251c	1.772±0.660c	16.962±8.148b	0.585±0.047c	2.464±0.194c	1.067±0.047c	0.104±0.027c	2.603±0.617c	0.394±0.057c
BrFLS2	Bra038647	1.000±0.000b	3.683±1.288a	0.048±0.005c	0.360±0.082bc	0.128±0.041bc	0.275±0.107bc	0.084±0.005c	0.110±0.051bc	0.000±0.000c	0.341±0.080bc	0.113±0.081bc	0.254±0.171bc
BrFLS3.1	Bra038648	1.000±0.000a	0.207±0.043c	0.000±0.000d	0.038±0.002d	0.051±0.031d	0.137±0.075cd	0.107±0.005cd	0.016±0.004d	0.528±0.198b	0.018±0.002d	0.058±0.024d	0.000±0.000d
BrFLS3.2	Bra029211	1.000±0.000cd	8.248±1.574a	0.599±0.101cd	4.319±0.602b	0.367±0.040d	1.104±0.023cd	0.210±0.000d	0.471±0.103d	0.000±0.000d	1.662±0.328c	0.579±0.149cd	0.093±0.016d
BrFLS3.3	Bra029212	1.000±0.000bcd	5.600±0.017a	1.256±0.115bc	5.444±0.857a	0.549±0.018de	1.275±0.268bc	0.361±0.306de	0.525±0.060de	0.087±0.057e	1.350±0.150b	0.635±0.197cde	0.686±0.149cde
BrFLS4	Bra037747	0.000±0.000c	1.000±0.000a	0.000±0.000c	0.116±0.040bc	0.000±0.000c	0.029±0.015c	0.094±0.133bc	0.000±0.000c	0.000±0.000c	0.177±0.126b	0.043±0.031c	0.031±0.044c
**Late biosynthetic genes (LBGs)**													
BrDFR1	Bra027457	1.000±0.000e	0.128±0.041e	0.210±0.031e	0.030±0.012e	12.383±2.489e	10.776±1.081e	77.429±7.546d	198.331±3.465c	4.832±1.397e	260.834±53.448b	223.341±35.300bc	340.821±6.441a
BrDFR2	Bra019062	0.000±0.000b	1.000±0.000a	0.000±0.000b	0.000±0.000b	0.141±0.199b	0.182±0.258b	0.000±0.000b	0.000±0.000b	0.000±0.000b	0.000±0.000b	0.259±0.367b	0.000±0.000b
BrDFR3	Bra010535	1.000±0.000a	0.021±0.021c	0.000±0.000c	0.006±0.001c	0.002±0.001c	0.009±0.003c	0.000±0.000c	0.000±0.000c	0.000±0.000c	0.000±0.000c	0.000±0.000c	0.608±0.240b
BrANS1	Bra013652	0.000±0.000d	1.000±0.000d	4.838±0.709d	0.938±0.202d	4.395±1.335d	18.782±4.073d	80.509±12.645d	864.017±34.760b	0.000±0.000d	635.670±57.933c	935.003±57.734b	1841.040±138.592a
BrANS2	Bra019	1.000±0.	1.305±0.	0.444±0.	0.637±0.	0.376±0.13	0.688±0.20	0.340±0.31	1.650±0.26	0.000±0.00	4.313±0.83	3.337±0.23	5.112±0.34

	350	000def	090de	169fg	022efg	8fg	5efg	1fg	6d	0g	4b	0c	6a
BrANS3	Bra017132	0.000±0.000b	1.000±0.000a	0.659±0.507ab	0.109±0.006b	0.000±0.000b	0.000±0.000b	0.868±1.227ab	0.525±0.371ab	0.000±0.000b	0.000±0.000b	0.000±0.000b	0.000±0.000b
BrANS4	Bra000045	6.799±0.000a	1.000±0.000def	0.988±0.137def	1.825±0.242c	0.000±0.000f	0.232±0.019ef	0.000±0.000f	1.248±0.394de	0.000±0.000f	0.000±0.000f	2.816±1.525b	0.000±0.000f
BrUF3GT1(BrUGT78D2()	Bra023594	1.000±0.000f	3.988±0.260c	1.918±0.082e	0.931±0.096f	4.042±0.248c	5.630±0.487b	0.171±0.012g	2.453±0.114de	0.444±0.199fg	6.530±0.584a	2.798±0.371d	2.278±0.613de
BrUF3GT2(BrUGT79B1)	Bra003021	1.000±0.000d	0.040±0.007d	0.163±0.036d	0.012±0.001d	0.900±0.284d	2.069±0.047d	1.795±0.187d	22.424±1.129c	2.185±2.189d	57.515±4.619b	25.536±1.477c	74.482±3.373a
BrUF5GT(BrUGT75C1()	Bra038445	1.000±0.000ef	0.073±0.021f	0.050±0.015f	0.026±0.006f	0.922±0.036ef	0.759±0.058ef	0.054±0.005f	2.980±0.291cd	1.729±0.606de	6.996±0.571b	3.771±0.390c	10.907±2.220a
Br5MAT	Bra036208	1.000±0.000ef	0.345±0.205f	0.232±0.015f	0.132±0.000f	4.169±1.795def	12.195±2.403def	13.226±5.620de	14.671±4.518d	5.315±0.404def	96.876±11.121b	62.603±11.146c	161.535±6.911a
Brp-CouT	Bra030550	1.000±0.000f	0.053±0.016f	0.340±0.074f	0.024±0.003f	6.701±0.722ef	11.826±0.236e	11.181±0.942e	50.624±5.598c	0.639±0.017f	78.038±4.247b	25.659±3.320d	93.834±8.774a
BrSAT	Bra012153	1.000±0.000f	74.882±16.672a	2.287±0.271f	11.993±0.710ef	6.549±0.846ef	50.336±4.265b	0.291±0.093f	17.542±2.060de	6.002±1.330ef	38.645±2.904c	27.600±2.977d	0.431±0.047f
BrUGT84A1.1	Bra039547	1.000±0.000a	0.016±0.006e	0.010±0.002e	0.015±0.007e	0.096±0.020cde	0.045±0.012de	0.156±0.101c	0.129±0.066cd	0.659±0.047b	0.034±0.010de	0.051±0.031de	0.087±0.045de
BrUGT84A1.2	Bra012784	1.000±0.000a	0.172±0.029c	0.076±0.010de	0.053±0.009e	0.172±0.033c	0.060±0.007e	0.019±0.004e	0.015±0.001e	0.691±0.084b	0.020±0.002e	0.035±0.004e	0.131±0.019cd
BrUGT84A2.1	Bra031290	1.000±0.000a	0.039±0.022bcd	0.014±0.010d	0.005±0.004d	0.074±0.055bc	0.027±0.017cd	0.090±0.042b	0.034±0.023cd	0.000±0.000d	0.017±0.008d	0.035±0.022cd	0.028±0.013cd
BrUGT84A2.2	Bra023872	1.000±0.000e	7.153±0.363d	1.731±0.141e	20.347±4.369b	8.931±0.000cd	7.637±1.018d	3.182±0.014e	11.229±1.046c	3.054±0.000e	1.000±0.016e	39.123±0.000a	0.316±0.066e
BrUGT84A3.1	Bra039545	1.000±0.000c	0.437±0.081de	0.704±0.158cd	0.024±0.009e	0.454±0.079de	0.068±0.003e	0.000±0.000e	0.041±0.000e	0.551±0.000de	2.205±0.261b	3.907±0.475a	0.558±0.251d
BrUGT84A3.2	Bra039544	1.000±0.000b	0.734±0.141b	0.783±0.431b	0.124±0.055b	0.143±0.006b	0.065±0.049b	5.026±3.215a	0.190±0.135b	1.613±0.737b	1.582±0.699b	1.126±0.514b	0.437±0.441b

Positive regulators

Independent R2R3-MYB regulatory genes

BrMYB12.1	Bra004456	1.000±0.000ab	0.000±0.000d	0.226±0.007d	0.062±0.018d	0.178±0.069d	0.095±0.006d	0.000±0.000c	1.091±0.093a	0.796±0.094b	0.131±0.000d	0.768±0.190b	0.517±0.296c

BrMYB12.2	Bra000453	1.000±0.000ab	1.037±0.252ab	0.339±0.093c	0.534±0.088bc	0.702±0.276abc	0.241±0.062c	0.288±0.053c	0.226±0.147c	0.159±0.062c	0.655±0.036abc	1.253±0.754a	0.497±0.395bc
BrMYB111.1	Bra037419	1.000±0.000c	0.005±0.000f	0.570±0.125de	0.024±0.001f	1.472±0.123b	0.364±0.061ef	3.799±0.584a	0.235±0.051ef	0.400±0.065def	0.044±0.022f	0.769±0.035e	0.198±0.094ef
BrMYB111.2	Bra036145	1.000±0.000a	0.050±0.007de	0.000±0.000e	0.006±0.004e	0.120±0.070de	0.028±0.020de	0.000±0.000e	0.000±0.000e	0.624±0.000b	0.085±0.039de	0.331±0.224c	0.181±0.028d

R2R3-MYB regulatory genes participate in MBW complex formation

BrMYB1	Bra001917	1.000±0.000f	2.597±0.324bc	0.606±0.148f	0.558±0.237f	4.732±0.285a	1.320±0.008de	1.404±0.039def	1.606±0.864de	2.122±0.699bcd	2.911±0.610b	1.949±0.382cde	4.143±0.270a
BrMYB2	Bra004162	1.000±0.000de	0.029±0.004e	0.164±0.027e	0.068±0.004e	0.757±0.004e	0.672±0.018e	2.075±0.085de	20.742±4.110b	3.018±0.490de	5.000±0.983cd	7.052±0.279c	35.183±4.714a
BrPAP1	Bra039763	1.000±0.000ef	0.416±0.115f	0.318±0.028f	0.238±0.032f	5.161±0.296b	0.376±0.019f	6.510±1.572a	0.892±0.027ef	2.154±0.403cd	2.055±0.318cde	2.216±0.428c	1.053±0.312def
BrTT2	Bra035532	1.000±0.000a	0.000±0.000c	0.000±0.000c	0.000±0.000c	0.000±0.000c	0.000±0.000c	0.000±0.000c	0.000±0.000c	0.000±0.000c	0.026±0.036b	0.000±0.000c	0.000±0.000c

bHLH regulatory genes

BrTT8	Bra037887	0.000±0.000c	0.000±0.000e	1.000±0.000e	1.769±0.281e	14.372±0.792cde	11.700±2.775de	0.000±0.000e	102.129±13.763b	49.273±4.714c	39.260±14.520cd	171.901±30.560a	132.482±41.830b
BrEGL3-1	Bra027653	1.000±0.000a	0.590±0.069bc	0.038±0.003f	0.206±0.060de	0.460±0.047cde	0.873±0.104ab	0.522±0.025cd	1.062±0.439a	0.267±0.063def	0.179±0.055ef	0.093±0.009f	0.557±0.217bc
BrEGL3-2	Bra027796	1.000±0.000a	0.033±0.008e	0.038±0.006e	0.044±0.004e	0.020±0.005e	0.101±0.005d	0.200±0.025b	0.084±0.020d	0.043±0.023e	0.036±0.006e	0.132±0.017c	0.092±0.011d
BrGL3	Bra025508	1.000±0.000a	0.056±0.010c	0.042±0.013c	0.037±0.004c	0.035±0.022c	0.034±0.008c	0.100±0.002c	0.140±0.024c	0.399±0.261b	0.095±0.026c	0.066±0.019c	0.112±0.013c

WD40 regulatory genes

BrTTG1	Bra009770	1.000±0.000a	0.300±0.042bc	0.134±0.013d	0.125±0.036d	0.137±0.008d	0.148±0.020d	0.379±0.157b	0.335±0.046b	0.095±0.016d	0.319±0.011b	0.267±0.035b	0.191±0.040cd

Negative regulators

Single-Repeat R3 MYB regulatory genes

BrCPC1	Bra004539	1.000±0.000f	17.276±1.904c	3.306±0.576de	21.069±1.317c	3.843±0.167de	19.930±2.096c	1.381±0.677f	59.863±6.975a	1.182±0.000f	18.675±0.285c	7.991±0.842d	27.221±0.271b
BrCPC2	Bra039283	1.000±0.000ef	2.492±0.378d	0.434±0.078fg	3.593±0.063c	0.504±0.147fg	2.735±0.090d	0.049±0.000g	7.524±0.364a	0.146±0.000g	2.823±0.194d	1.132±0.086e	4.177±0.760b
BrMYBL2.1	Bra016164	1.000±0.000d	62.517±6.230	64.453±4.222	110.757±3.51	0.533±0.154d	2.345±0.734d	0.000±0.000d	2.641±0.369d	0.000±0.000d	4.338±0.555cd	8.739±3.445c	1.834±0.082d

			b	b	8a								
BrMYBL2.2	Bra007957	1.000±0.000bcd	6.123±0.665a	0.127±0.014cd	0.516±0.178cd	5.813±0.521a	4.711±0.859a	0.000±0.000d	0.456±0.217cd	2.293±0.529b	1.708±0.473bc	5.903±1.944a	0.368±0.121cd
**LATERAL ORGAN BOUNDARY DOMAIN (LBD) genes**													
BrLBD37.1	Bra012164	1.000±0.000e	0.706±0.082f	2.062±0.217a	1.298±0.026cd	1.407±0.099c	0.584±0.050fg	1.627±0.150b	1.156±0.069de	0.500±0.012gh	0.356±0.031h	0.509±0.038gh	0.168±0.012h
BrLBD37.2	Bra031833	1.000±0.000c	1.207±0.220b	1.094±0.129bc	0.494±0.028d	1.774±0.080a	0.492±0.059d	0.035±0.025ef	0.044±0.014ef	0.000±0.000f	0.206±0.037e	1.165±0.115b	0.037±0.003ef
BrLBD37.3	Bra037847	1.000±0.000efg	4.687±0.751a	1.701±0.426de	2.155±0.868cd	2.724±0.489bc	3.220±0.409b	0.000±0.000h	0.134±0.060gh	0.648±0.252fgh	0.694±0.263fgh	1.579±0.196def	0.390±0.120gh
BrLBD38.1	Bra036040	1.000±0.000e	13.345±2.193b	4.038±0.506d	7.678±0.350c	0.796±0.119e	2.976±0.201d	0.133±0.012e	0.751±0.154e	2.894±0.628d	14.929±1.799b	18.185±0.783a	3.515±0.508d
BrLBD38.2	Bra012913	1.000±0.000def	4.059±0.774c	4.810±0.475b	5.753±0.446a	0.204±0.055g	1.170±0.147d	0.058±0.000g	0.378±0.037fg	0.103±0.103g	1.106±0.231de	0.383±0.116fg	0.451±0.038fg
BrLBD39.1	Bra011772	1.000±0.000de	5.954±0.554b	0.453±0.007e	1.564±0.167c	1.001±0.169de	1.585±0.158d	0.059±0.026e	0.095±0.004e	0.645±0.063de	13.379±1.449a	0.461±0.009e	0.346±0.067e
BrLBD39.2	Bra017831	1.000±0.000cd	4.969±0.682a	0.238±0.074e	1.202±0.079cd	0.201±0.037e	1.095±0.064cd	0.092±0.003e	0.298±0.038e	0.000±0.000e	2.593±0.377b	0.379±0.133e	1.602±0.295c
**Transport genes**													
BrGST1	Bra008570	1.000±0.000e	0.449±0.267e	0.935±0.447e	0.106±0.032e	17.672±1.724e	35.940±5.354e	458.523±7.313c	242.155±27.210d	13.146±2.859e	863.941±120.297ab	720.475±263.406b	991.138±132.289a
BrGST2	Bra023602	1.000±0.000d	0.244±0.057d	2.272±0.334d	0.088±0.032d	11.830±1.920d	23.583±1.387d	186.722±44.120bc	75.274±3.095cd	10.093±1.017d	544.676±73.176a	244.167±109.476b	563.271±125.756a

Table S2. Genes involved in anthocyanin biosynthesis in *B. rapa*.

Chromosome	Start	Stop	*At Name*	*At* Gene	*Br Name*	*Br* Gene	Annotation
A05	4098130	4100879	*PAL1*	AT2G37040	*BrPAL1.1*	Bra005221	Phenylalanine ammonia-lyase 1
A04	16132008	16134574	*PAL1*	AT2G37040	*BrPAL1.2*	Bra017210	Phenylalanine ammonia-lyase 1
A09	28184757	28187233	*PAL2*	AT3G53260	*BrPAL2.1*	Bra006985	Phenylalanine ammonia-lyase 2
A04	3961512	3964566	*PAL2*	AT3G53260	*BrPAL2.2*	Bra039777	Phenylalanine ammonia-lyase 2
A07	14754085	14756786	*PAL2*	AT3G53260	*BrPAL2.3*	Bra003126	Phenylalanine ammonia-lyase 2
A02	625387	626594	*PAL3*	AT5G04230	*BrPAL3.1*	Bra028793	Phenylalanine ammonia-lyase 3
A04	9481038	9483492	*PAL3*	AT5G04230	*BrPAL3.2*	Bra030322	Phenylalanine ammonia-lyase 3
A05	22819640	22826303	*PAL4*	AT3G10340	*BrPAL4*	Bra029831	Phenylalanine ammonia-lyase 4
A05	7378464	7380483	*C4H*	AT2G30490	*BrC4H1*	Bra018311	Cinnamate-4-hydroxylase
A04	13681735	13687441	*C4H*	AT2G30490	*BrC4H2*	Bra021636	Cinnamate-4-hydroxylase
A04	13688684	13690602	*C4H*	AT2G30490	*BrC4H3*	Bra021637	Cinnamate-4-hydroxylase
A03	7147092	7149367	*C4H*	AT2G30490	*BrC4H4*	Bra022802	Cinnamate-4-hydroxylase
A03	7151974	7153871	*C4H*	AT2G30490	*BrC4H5*	Bra022803	Cinnamate-4-hydroxylase
A05	11246278	11249002	*4CL1*	AT1G51680	*Br4CL1.0*	Bra030429	4-coumarate:CoA ligase 1
A05	17306583	17311892	*4CL2*	AT3G21240	*Br4CL2.1*	Bra031262	4-coumarate:CoA ligase 2
A05	17296799	17300456	*4CL2*	AT3G21240	*Br4CL2.2*	Bra031263	4-coumarate:CoA ligase 2
A05	17276121	17280811	*4CL2*	AT3G21240	*Br4CL2.3*	Bra031265	4-coumarate:CoA ligase 2
A05	17255035	17257878	*4CL2*	AT3G21240	*Br4CL2.4*	Bra031266	4-coumarate:CoA ligase 2
A07	20172546	20177374	*4CL3*	AT1G65060	*Br4CL3*	Bra004109	4-coumarate:CoA ligase 3
A03	18663880	18669799	*4CL4*	AT3G21230	*Br4CL4.1*	Bra001819	4-coumarate:CoA ligase 5
A03	18675088	18678049	*4CL4*	AT3G21230	*Br4CL4.2*	Bra001820	4-coumarate:CoA ligase 5
A10	12655973	12657235	*CHS*	AT5G13930	*BrCHS1*	Bra008792	Chalcone synthase
A03	2596137	2597594	*CHS*	AT5G13930	*BrCHS2*	Bra006224	Chalcone synthase
A02	2357734	2359185	*CHS*	AT5G13930	*BrCHS3*	Bra023441	Chalcone synthase
A09	695971	697415	*CHS*	AT5G13930	*BrCHS4*	Bra036307	Chalcone synthase
A02	23204660	23205928	*CHS*	AT5G13930	*BrCHS5*	Bra020688	Chalcone synthase
A09	29055564	29057157	*CHI*	AT3G55120	*BrCHI1*	Bra007142	Chalcone isomerase
A07	15173091	15175119	*CHI*	AT3G55120	*BrCHI2*	Bra003209	Chalcone isomerase
A03	30108206	30109397	*CHI*	AT3G55120	*BrCHI3*	Bra017728	Chalcone isomerase

A09	27095567	27097080	*F3H*	AT3G51240	*BrF3H1*	Bra036828	Flavanone 3-hydroxylase
A01	15030232	15032156	*F3H*	AT3G51240	*BrF3H2*	Bra029996	Flavanone 3-hydroxylase
A03	21908585	21910045	*F3H*	AT3G51240	*BrF3H3*	Bra012862	Flavanone 3-hydroxylase
A10	14356094	14358845	*F3'H*	AT5G07990	*BrF3'H*	Bra009312	Flavonoid 3'-hydroxylase
A10	14152996	14154560	*FLS1*	AT5G08640	*BrFLS1*	Bra009358	Flavonol synthase 1
A06	14983444	14986024	*FSL2*	AT5G63580	*BrFLS2*	Bra038647	Flavonol synthase 2
A06	14996259	14997537	*FLS3*	AT5G63590	*BrFLS3.1*	Bra038648	Flavonol synthase 3
A02	25978824	25980695	*FLS4*	AT5G63595	*BrFLS3.2*	Bra029211	Flavonol synthase 4
A02	25974752	25976735	*FLS5*	AT5G63600	*BrFLS3.3*	Bra029212	Flavonol synthase 5
A09	3988206	3991271	*FLS5*	AT5G63600	*BrFLS4*	Bra037747	Flavonol synthase 5
——	——	——	*MYB11*	AT3G62610	——	——	MYB Domain Protein 11
A05	222923	225991	*MYB12*	AT2G47460	*BrMYB12.1*	Bra004456	MYB Domain Protein 12
A03	11210760	11213776	*MYB12*	AT2G47460	*BrMYB12.2*	Bra000453	MYB Domain Protein 12
A06	19963815	19966634	*MYB111*	AT5G49330	*BrMYB111.1*	Bra037419	MYB Domain Protein 111
A09	2246093	2248814	*MYB111*	AT5G49330	*BrMYB111.2*	Bra036145	MYB Domain Protein 111
A09	10926334	10927890	*DFR*	AT5G42800	*BrDFR1*	Bra027457	Dihydroflavonol-4-reductase
A03	26526178	26528474	*DFR*	AT4G27250	*BrDFR2*	Bra019062	Dihydroflavonol-4-reductase
A08	14247307	14248713	*DFR*	AT4G35420	*BrDFR3*	Bra010535	Dihydroflavonol-4-reductase
A01	6885692	6887113	*ANS (LDOX)*	AT4G22880	*BrANS1*	Bra013652	Leucoanthocyanidin dioxygenase
A03	24796232	24797395	*ANS (LDOX)*	AT4G22880	*BrANS2*	Bra019350	Leucoanthocyanidin dioxygenase
A04	16549604	16551604	*ANS (LDOX)*	AT2G38240	*BrANS3*	Bra017132	Leucoanthocyanidin dioxygenase
A03	9021983	9024777	*ANS (LDOX)*	AT2G38240	*BrANS4*	Bra000045	Leucoanthocyanidin dioxygenase
A10	6063162	6064651	*UGT79B1*	AT5G54060	*BrUGT79B1(BrUF3GT2)*	Bra003021	Anthocyanin 3-O-glucoside: 2-O-xylosyltransferase
——	——	——	*UGT79B1*	AT5G54060	*BrUGT79B1.2*	Bra035004	Anthocyanin 3-O-glucoside: 2-O-xylosyltransferase
A08	8755970	8757334	*UGT75C1*	AT4G14090	*BrUGT75C1(BrUF5GT)*	Bra038445	Anthocyanin 5-O-glucosyltransferase
A02	3087246	3088800	*UGT78D2*	AT5G17050	*BrUGT78D2(BrUF3GT1)*	Bra023594	Anthocyanidin 3-O-glucosyltransferase
A09	1925788	1927140	*5MAT*	AT3G29590	*Br5MAT*	Bra036208	Malonyl-CoA:anthocyanidin 5-O-glucoside-6"-O-malonyltransferase
A01	11563809	11565239	*UGT84A1*	AT4G15480	*BrUGT84A1.1*	Bra039547	Sinapic acid:UDP-glucose glucosyltransferase
A03	22311911	22313383	*UGT84A1*	AT4G15480	*BrUGT84A1.2*	Bra012784	Sinapic acid:UDP-glucose glucosyltransferase
A05	17020229	17021722	*UGT84A2*	AT3G21560	*BrUGT84A2.1*	Bra031290	Sinapic acid:UDP-glucose glucosyltransferase
A01	19817667	19819160	*UGT84A2*	AT3G21560	*BrUGT84A2.2*	Bra023872	Sinapic acid:UDP-glucose glucosyltransferase

A01	11553894	11555345	*UGT84A3*	AT4G15490	*BrUGT84A3.1*	Bra039545	Sinapic acid:UDP-glucose glucosyltransferase
A01	11534241	11534960	*UGT84A3*	AT4G15490	*BrUGT84A3.2*	Bra039544	Sinapic acid:UDP-glucose glucosyltransferase
A08	20600205	20602987	*UGTp-CouT*	AT1G03940	*Brp-CouT*	Bra030550	HXXXD-type acyl-transferase family protein
A07	11924527	11938309	*SCPL*	AT2G23000	*BrSAT*	Bra012153	Sinapoyl-Glc:anthocyanin acyltransferase
A02	8839008	8840737	*PAP1(MYB75)*	AT1G56650	*BrPAP1*	Bra039763	Production of Anthocyanin Pigment 1
A03	19357364	19358849	*PAP1(MYB75)*	AT1G56650	*BrMYB1*	Bra001917	Production of Anthocyanin Pigment 1
A07	20426416	20431671	*PAP1(MYB75)*	AT1G56650	*BrMYB2*	Bra004162	Production of Anthocyanin Pigment 1
A07	20426416	20431671	*PAP2(MYB90)*	AT1G66390	*BrMYB2*	Bra004162	Production of Anthocyanin Pigment 2
A03	19357364	19358849	*PAP2(MYB90)*	AT1G66390	*BrMYB1*	Bra001917	Production of Anthocyanin Pigment 2
A02	8839008	8840737	*PAP2(MYB90)*	AT1G66390	*BrPAP1*	Bra039763	Production of Anthocyanin Pigment 2
A09	15769736	15773288	*TT8*	AT4G09820	*BrTT8*	Bra037887	Transparent Testa 8
A04	8626979	8631281	*GL3*	AT5G41315	*BrGL3*	Bra025508	Glabrous 3
A09	6529998	6533684	*EGL3*	AT1G63650	*BrEGL3.2*	Bra027796	Enhancer of Glabrous 3
A09	7531679	7534777	*EGL3*	AT1G63650	*BrEGL3.1*	Bra027653	Enhancer of Glabrous 3
A06	17739539	17740552	*TTG1*	AT5G24520	*BrTTG1*	Bra009770	Transparent Testa Glabrous 1
A02	24761813	24762175	*TTG1*	AT5G24520	*BrTTG1.2*	Bra029411	Transparent Testa Glabrous 1
A07	22386380	22387236	*MYBL2*	AT1G71030	*BrMYBL2.1*	Bra016164	MYB-Like 2
A02	11125340	11126192	*MYBL2*	AT1G71030	*BrMYBL2.2*	Bra007957	MYB-Like 2
A05	706175	706817	*CPC*	AT2G46410	*BrCPC1*	Bra004539	CAPRICE
A04	18882042	18882818	*CPC*	AT2G46410	*BrCPC2*	Bra039283	CAPRICE
A07	11864765	11865628	*LBD37*	AT5G67420	*BrLBD37.1*	Bra012164	LOB Domain-containing Protein 37
A02	26735230	26736023	*LBD37*	AT5G67420	*BrLBD37.2*	Bra031833	LOB Domain-containing Protein 37
A09	4481055	4481859	*LBD37*	AT5G67420	*BrLBD37.3*	Bra037847	LOB Domain-containing Protein 37
A09	26375754	26376800	*LBD38*	AT3G49940	*BrLBD38.1*	Bra036040	LOB Domain-containing Protein 38
A03	21565949	21566767	*LBD38*	AT3G49940	*BrLBD38.2*	Bra012913	LOB Domain-containing Protein 38
A01	631422	632295	*LBD39*	AT4G37540	*BrLBD39.1*	Bra011772	LOB Domain-containing Protein 39
A03	30822548	30823423	*LBD39*	AT4G37540	*BrLBD39.2*	Bra017831	LOB Domain-containing Protein 39
A10	11677671	11678470	*TT19*	AT5G17220	*BrTT19.1(BrGST1)*	Bra008570	Transparent Testa 19
A02	3117740	3118547	*TT19*	AT5G17220	*BrTT19.2(BrGST2)*	Bra023602	Transparent Testa 19
A08	8305228	8306174	*TT2*	AT5G35550	*BrTT2*	Bra035532	Transparent Testa 2
A09	225267	229473	*ANL2*	AT4G00730	*BrANL2*	Bra037355	ANTHOCYANINLESS 2

A09	3619 7266	3619 8532	*AN11*	AT1G 12910	*BrAN11*	Bra02 6947	ANTHOCYANIN11

The information of this table was collected from *Brassica* database. *At*, *Arabidopsis*; *Br*, *Brassica*.

Table S3. Primers for the qRT-PCR analysis in Chinese Cabbages.

Gene	Forward Primer (5' to 3')	Reverse Primer (5' to 3')	Product (bp) a
BrEF-1α	ATACCAGGCTTGAGCATACCG	GCCAAAGAGGCCATCAGACAA	117
BrPAL1.1	TTAGGAAGCCTGTGGTGAATC	GTGCCTTTGCCCATACTCTC	163
BrPAL1.2	TACGGTTTCAAAGGAGCG	TGATGTCTTGCGAGATGAGATTAG	150
BrPAL2.1	AACTCCAAAGCCACAGGTCC	TCAGGCTTCCCGCTCATAA	236
BrPAL2.2	CTCCGTGGAACCATAACC	GGCGAGGACCGATTGAAC	264
BrPAL2.3	GGGTCGCATTGCAGACAGA	GTTATGGTTCCACGGAGGG	229
BrPAL3.1	ATTCCTCAACCACGACATCA	GCTCGTCTCGCACAAACCT	275
BrPAL3.2	GAGCAAAGGGACGACGGT	ATCCAGTGGTGATTCCATAAGT	119
BrPAL4	ACTGCCTCCGATGCCTTTA	ACAGAACCATCGAAGCCAGAC	121
BrC4H1	CGGTCCTATGCCGATTCCA	ACCATGTCCTGCCCTTTGC	249
BrC4H2	CGCTGCGATTGAGACAACACT	TCCTTAGCTTGCTCTGGATTT	86
BrC4H3	CGAACACTGGCGTAAGATGC	TCACGTCCTCCACGACACTC	116
BrC4H4	TGTTATGTTCGGTAAAAGGTTCG	GTAGGCTTAGCACTCGCAATC	246
BrC4H5	CTCTTCCCCAAACCTAACCAA	GTTTCCTCAACACGATCCCTTT	287
Br4CL1	GAGTTGGTGCCTCCATCTTG	CTTCCGTCATTCCGTATCCCT	264
Br4CL2.1	CGACAAACTGAAAAGCCTCC	CGGTGAAGTTGACGTTAGGG	272
Br4CL2.2	GACCTGACTCAAGCGGACGA	CTGGTGAAGTTGAGATTAGGGTTT	185
Br4CL2.3	TCACCTACGCCGATGTTCA	GGCTTGTGCCTGTTTTGC	209
Br4CL2.4	CCACAGCAGCAACGATAGAC	CAACAGGAACCTCACCAGCAT	228
Br4CL3	ATCGGGGCGTTATTGGA	GAGCCTGATTTTGACGGTGTT	209
Br4CL4.1	GGCTCAAGCGGACGAAAC	TGTGAAACATAGGGAGGAAA	218
Br4CL4.2	TCACCACAACCGCCAATC	CCACGCAAACTATCAGAACG	147
BrCHS1	CGCTCATTGTCGGTTCG	AGGGAGTTCCAGTCGCTTAT	235
BrCHS2	CTGAGAACAACCGTGGCG	CGAGTCGGGAAGAATGG	221
BrCHS3	CTACTACTTCCGCATCACCAAC	GTGGCGTTTTCTTATGGTCG	94
BrCHS4	AAAGAGGCGGCAGTGAAG	GGCGAGACGGAGGACAGT	204
BrCHS5	TACCATCAAGGCTGCTACGC	TCAGGGTCTGAACCCACAAT	209
BrCHI1	CCTCCTGGTGCTTCCATCCT	GAACTGCCTCTGCCAACAACT	130
BrCHI2	CACTGTCCAACACCGTTACCC	ACACCGATGACCGTGAAGAT	167
BrCHI3	ATCCGTCCCTTTCTTCCG	ACTGCTTTAGCCTCAGAGTATGTG	168
BrF3H1	AAGCGGATACACGGTTGC	ACACGAGACGTAGGGAAG	289
BrF3H2	GTCAACTACTATCCCGAGTGTCC	GCTGCCTAATATGTCGTCCTG	108
BrF3H3	ATTACCCCAAATGCCCTCA	GAACCTCCCGTTGCTCAGATAG	209
BrF3'H	TGCCCGCACTTGATTGTTT	CCGTTCTTCATCGCCTCGT	118
BrFLS1	ACTCTCGGAAGGGTTAGGCT	CGATGTGAACAATGACGGC	254
BrFLS2	ATCTTTTCCACAGAATCTTGCC	CTCCGACTTTTCGCCACC	200
BrFLS3.1	TACTATCCGCCGTGTCCTG	GAACGCCTGAAGCCCTAA	111
BrFLS3.2	TTTTCTATCCGCCGTGTCC	TACCTCCCATTGCTCATCC	205
BrFLS3.3	CGTGCGGTGGTAAAAGCC	CCTTCTCAGGCAGCTCAAAGA	124
BrFLS4	ACTACTATCCGCCGTGTCGTC	TTCTCCGTGTTCAAAGTCGTTC	247
BrMYB12.1	AGCCAGAGGGTCATCAGAGA	AGCCTCTTTCTCATCGCAAA	187
BrMYB12.2	GTCTTCAGTCTTGTCCGTCG	AATGTTATTGTCATCCCCTTG	125
BrMYB111.1	CAATGTCTCCCACAACCTAAA	CCAACCATTCATACGCTCCC	100
BrMYB111.2	TGTTATGTGCGTTTGTTTGAGG	CGATCCACTTGCCGTATTTCT	278
BrDFR1	GACGGCGTTTTCCACATAG	TCCCCAACACTCCATTCAC	94
BrDFR2	AGGAAGGAAAAGCGACGAC	AGATTAACCTGATCCGGTGAGA	286
BrDFR3	GATGTCCTCGGGCTGCTAA	CGAAGTGTCCAAGTCGTAATGC	258

BrANS1	GAAGACGAAACCATCCCGTGAG A	TTAGCCAACTTACTTCCATACC	220
BrANS2	CCAAGGGTATGGAAGTAAAT	TAAGCCGAGACCGACAGAG	214
BrANS3	GGAGCATCAGGTGGTCGTT	GGTTGGTCTTCGGTTAGTAGTTC	117
BrANS4	ACGCAAACTCACCCGACA	GCCCCGACTTTATCTTCTCC	286
BrUGT79B1(BrUF3GT 2)	CCCTAACTCTTGTCCCTGCTG	CAATGCCCTCGTGTCTCC	165
BrUGT75C1(BrUF5GT)	ATCGGACGAGGACTACATC	CCACCACCAGACCTTTATC	233
BrUGT78D2(BrUF3GT 1)	AACTCCTCGTTATTCCCCTCAG	CTGTCCAAAACGCAACCCA	271
Br5MAT	CTTTCTTTGACTTGGGTTGGC	CGGCGAGTGGGAGATAATG	144
BrUGT84A1.1	TCTTCAGTGGCGTGTTTTGTC	TTCTCCGCTATGTCCTCCC	206
BrUGT84A1.2	TCCTTCAGTGGCGTGTTTC	TCCCCATTGCGGACAAC	97
BrUGT84A2.1	GACCGAGCCCTCAAACCTATG	GGCAAGCACAAGACTGGACC	286
BrUGT84A2.2	CATTCGGAACCGTCGCTTAT	CTCCCCACTGAGGCAAACA	297
BrUGT84A3.1	GGTTAGCCGACGATGACGA	CGCAAGACTGGACCCATAGAA	219
BrUGT84A3.2	CCTTCTTATGGGTGGTTCGG	AGCCTCTACGGTTGAGTTCC	179
Brp-CouT	TCAAAGACTATCTCCCTAACCTC A	ACTCAGCGACTGTGAAAAGAA	156
BrSAT	CAGCATGTTGATTCTGGCTCT	CCTCTTTCTCACCAATACCGA	103
BrPAP1	TTCCTTGCTCTTCAACCCA	GCTCATATTTCTCCGCATCTTT	201
BrMYB1	GGAAACAGGTGGTCTTTAATTG	GTTTCTTGCTCAGATGGGTG	91
BrMYB2	GTCAGAATAGGTGGTGGGAAAG T	CCATCCAACAGGCTCCAAAGT	133
BrTT8	AAAGCGTATGCGAGGAG	TGTTTGACTTCGGGTGGTT	154
BrGL3	CCGCATCAATCGTAGCAC	AACCGTTCATTCAGTTTCTC	169
BrEGL3.2	ATCTAAAGAAACACCTCGCAG	CCATCTCCCCATTCCAGC	106
BrEGL3.1	CTCAGACGTTTGTTGGAGCA	CGAGATCACGCCTTGGTAAT	137
BrTTG1	ATCCTATGTCGGTTTACTCGG	ACGCAATGCCAATCCAAT	84
BrMYBL2.1	ACCGACGACGAAGTAAGGAT	GGGACGAGACCGATGTTGAG	290
BrMYBL2.2	CCAGGTTGCTACATCGTGC	TTATTTCAGTATCGGTTCGTCC	183
BrCPC1	CGTGTTCCGAAGAAGTAAGTAG C	CCGTGTTTCATAAGCCAATA	175
BrCPC2	ATGTTCCGAAGAAGTGAGTAGC	CGAATCCGTGTTTCATAAGC	179
BrLBD37.1	TTTCATCTCCGCTGTGCC	TGCTCCGTTGACTGGGTT	97
BrLBD37.2	GGGAGAACAGTGAATCCAGTC	GGCAGAAGAGTCCAGCGTAG	282
BrLBD37.3	AATCGGAATGCTGTGGACG	GCTTGAAAATCTTGAATGGTGG	226
BrLBD38.1	GCTTGTGGGAGAACTGTGAACC	GAGAATCGGCAGTGGTGGTAG	251
BrLBD38.2	CGGTTCTTTGAGACCTATCCC	TCTTGTTGTTGCTCTGACGCTA	212
BrLBD39.1	TCCACCGCCGCAGTTTA	CGCCGCTGATTCCATTTTC	101
BrLBD39.2	CATCCTTCGTCCTTGCTTCC	GCAGGGCGGTTTAGTTCAG	135
BrTT19.1(BrGST1)	GAGCCATTGCGAGATACTACG	CGACCTCAACATCAGCCCAC	111
BrTT19.2(BrGST2)	TAGTCATCAAGCCCAGGTTAGG	CGACGCAAGCCGATTCTC	107
BrTT2	AAACCAACCAACAGAAAAGTCG	TTCCACAACCCAAACCAT	276
BrANL2	TCCCCACTGGTCTAGCTTATG	CTCGGCTCCTCCTGTTCTTA	240
BrAN11	AGGAGCGGCGGTTTCTA	CTTTGTGGGAGGGTAAGGATG	293

a: sequence length was acquired according to the BRAD database.
